# Livestock Integration Into Cropping Systems Enhances Their Climate Change Resistance and Mitigation While Reducing Their Environmental Impacts

**DOI:** 10.1111/gcb.70765

**Published:** 2026-02-25

**Authors:** Mathieu Delandmeter, Bruno Basso, Xavier Fettweis, Christophe Lacroix, Pierre Aubry, Jérôme Bindelle, Benjamin Dumont

**Affiliations:** ^1^ Gembloux Agro‐Bio Tech, TERRA Teaching and Research Centre, Plant Sciences/Crop Science Liege University Gembloux Belgium; ^2^ Department of Earth and Environmental Sciences Michigan State University East Lansing Michigan USA; ^3^ W.K. Kellogg Biological Station Michigan State University Hickory Corners Michigan USA; ^4^ SPHERES Research Unit, Department of Geography Liege University Liège Belgium; ^5^ Gembloux Agro‐Bio Tech, TERRA Teaching and Research Centre, Animal Sciences Liege University Gembloux Belgium

**Keywords:** circularity, climate change, crop model, crop‐livestock, ecosystem services, greenhouse gas, human diet, leaching, resistance, STICS

## Abstract

The sustainability of cropping systems is linked to their circularity, which is their ability to close resource cycles such as carbon and nitrogen through strategies for managing crop residues, byproducts, and other inputs. Here, we investigate three crop rotations—business‐as‐usual (BAU), vegan, and integrated crop‐livestock systems (ICLS)—varying in livestock integration, crop residue fate, and human diet sustained. Under ten climate change scenarios, we compare their impacts on multiple ecosystem services during 24 years over 541,800 ha in Belgium using a validated crop model. All three circularity scenarios are found to be net greenhouse gas (GHG) emitters, with increasing intensity under climate change. The BAU system, favoring cash crops such as sugarbeet or potato, demonstrates the highest productivity, which, however, is highly variable across years and comes with greater environmental impacts such as GHG emissions (+45% and +23% compared to ICLS and Vegan in average—i.e., across all sites and climate scenarios). The Vegan system has lower carbon sequestration than the ICLS due to the lack of pasture and livestock, which, however, is partly offset by the regular incorporation of crop residues into the soil. Finally, ICLS, which include temporary pastures and sheep, demonstrate intermediate productivity levels compared to the other systems. However, they offer the greatest stability and resistance to extreme weather (+43% and +86% for stability compared to BAU and Vegan, in average), with better environmental performance. Therefore, our study reveals the benefits of crop‐livestock systems in terms of climate change adaptation, through stability and resistance to extreme climate events, and mitigation, through soil carbon sequestration and reduced greenhouse gas emissions and nitrate leaching. Moreover, our findings highlight the critical links between farm‐level circularity, soil‐crop feedbacks, human diet, and climate change.

## Graphical abstract

This study compares three types of farming systems in Belgium—conventional, vegan, and crop–livestock integrated—under future climate change. Using crop model 24‐year simulations over 541,800 ha under ten climate change scenarios, we assessed how productive, stable, and environmentally friendly they are over time. While conventional systems exhibit the highest productivity, they also cause higher environmental impacts and are less stable under extreme weather. Crop–livestock systems offer a good balance, with more stable productivity, lower greenhouse gas emissions, and better soil health. Overall, integrating crops and livestock helps farms adapt to climate change while reducing environmental impacts.

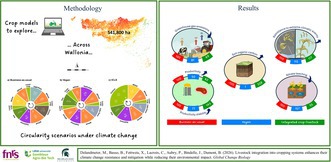

## 1 Introduction

Climate change impacts food quantity, quality, and safety (Myers et al. 2017; Mbow et al. 2019; Jägermeyr et al. 2021), notably through extreme climatic events such as droughts and extreme heat, which damage crop production (Lesk et al. 2016; Powell and Reinhard 2016). Yet climate change is also driven by greenhouse gas (GHG) emissions from food systems: they are responsible for approximately one‐third of global anthropogenic GHG emissions (Crippa et al. 2021). These emissions can be categorized into emissions from crop and livestock activities at the farm level (10%–14%), land use and land‐use changes related to agriculture (5%–14%), and food production and distribution, including fuel utilization and the manufacture of agricultural inputs such as chemical fertilizers (5%–10%) (Mbow et al. 2019). Moreover, climate change is expected to exacerbate the environmental impacts of agriculture, by affecting agricultural productivity, reducing the efficacity of agrochemicals and increasing their loss to the environment, and increasing crop pests and soil erosion (Yang et al. 2024).

Livestock production systems, in particular, are frequently criticized for their significant impacts, including deforestation, eutrophication of freshwater and marine ecosystems, biodiversity loss, and GHG emissions (Xu et al. 2021; Bidoglio et al. 2024). Linked to livestock production, human diets which are high in red meat are characterized as ‘lose‐lose’, that is, being environmentally unsustainable and unhealthy (Garnett 2016). Hence, the literature frequently advocates for reducing or even eliminating animal products from human diets as a sustainable strategy (e.g., Tilman and Clark 2014; Godfray et al. 2018; Poore and Nemecek 2018). However, there is no agreement on the extent to which animal‐source foods should be reduced to mitigate the environmental impacts of food systems (Frehner et al. 2020). The EAT‐Lancet Commission proposed a reference diet aimed at promoting health while respecting planetary boundaries (Willett et al. 2019). This diet emphasizes a variety of plant‐based foods while limiting or entirely excluding animal‐derived products. Yet, according to Van Selm et al. (2022), focusing on producing primarily milk, dairy beef, and pork (rather than poultry) within the EAT‐Lancet diet could further decrease GHG emissions and reduce arable land use, aligning with the findings of Poux and Aubert (2018).

More generally, animals play a key role in circular food systems, transforming byproducts, residues and grass resources, which are not suitable for human consumption, into valuable food (Van Zanten et al. 2019). They also contribute to nutrient cycling through feces, urine, and manure, enriching soils within individual plots but also promoting the use of nonfarming areas through the movement of animals (Bonaudo et al. 2014). Additionally, they offer a range of other benefits, such as controlling weeds, pests, and diseases, enhancing Net Primary Productivity (NPP), and promoting biodiversity (Soussana and Lemaire 2014; Franzluebbers and Martin 2022). In this direction, integrated crop‐livestock systems (ICLS) harness the complementary relationships between crops and livestock across various scales, from within a single farm—through practices like grazing on pastures or cover crops—to external cooperation between farmers—involving resource exchanges such as manure, grain, and straw (Martin et al. 2016). In the opposite, highly specialized systems have become the norm in Westernized agriculture, due to globalization, industrial development, and liberalization of trade (Garrett et al. 2020). They however rely on external inputs such as synthetic fertilizer produced with fossil fuel and high levels of pesticides to decouple crops and livestock (Liu et al. 2010).

The potential consequences of various agronomic strategies—such as maintaining highly specialized agricultural systems or eliminating livestock—remain poorly understood. Most research on agricultural circularity, investigating the optimal use of products (main outputs of agricultural systems—e.g., wheat grain), byproducts (secondary outputs produced during processing—e.g., wheat bran) and crop residues (leftover after harvest—e.g., wheat straw)—for food, feed, fibre or energy—relies on mass‐flow models. These models estimate inputs and outputs, like nitrogen fertilizers and crop yields, based on national statistics or surveys. They are particularly effective for assessing land use and GHG emissions across different levels of crop‐livestock integration (van Selm et al. 2023; Schader et al. 2015). However, they offer limited insight into broader ecosystem services, such as nitrate leaching reduction or resilience to extreme weather events, and do not easily consider particularities of pedoclimatic contexts. Addressing these complexities—especially in the context of climate change—requires a process‐based approach that focuses on climate‐soil‐crop management interactions, with particular attention to water, carbon and nitrogen cycles. Soil‐crop models effectively assess climate change impacts on crops (Asseng et al. 2015), and Basso et al. (2018) showed that it is primordial to account for soil feedbacks as SOC decline due to increased temperatures influences yields.

Our study covers half of Belgium, offering the particularity of very contrasting pedoclimatic conditions, and spanned ten historical and future climate scenarios. We compare three agronomic circularity scenarios, focusing on varying levels of livestock integration and crop residues use. These three scenarios are simulated with the soil‐crop model STICS (Beaudoin et al. 2022), that has been widely validated in the agronomic context of the study (Delandmeter et al. 2023 and 2024b). The model was run over 11,515 distinct locations that cover 541,800 ha in the Southern part of Belgium (Wallonia region), and under ten climate scenarios: historical (1980–2010) and future climate change conditions, offering contrasting temperature, rainfall and atmospheric CO_2_ patterns under three global warming scenarios (+2°C, +3°C and +4°C) simulated by three distinct Earth System Models (MPI, CMCC and MIR; see Methods). Here we demonstrate how varying degrees of livestock integration within cropping systems affect a broad spectrum of ecosystem services, that is, GHG emissions reduction (regulating service), soil organic carbon (SOC) sequestration (supporting service), nitrate leaching reduction (supporting and regulating service), and productivity and its stability and resistance to extreme climatic events (provisioning services).

## 2 Materials and Methods

### 2.1 Circularity Scenarios

We compared three circularity scenarios, based on the work of De Clerck et al. (2026). In the first step of this work, in a participatory approach with agronomy, nutrition and ecology experts, 40 different crop rotations were designed, varying in duration and in the composition of crop species and types. Good agricultural practices were followed and used as agronomic constraints to design the crop rotation. It includes principles such as alternating botanical families and crop types (e.g., spring vs. winter crops), maximizing the inclusion of cover crops, or guaranteeing a minimum return time between the same crop (e.g., 4 years between sugarbeet and potato, minimum 3 years between two legumes if species is changed, etc.). An innovative approach was then developed to assess the suitability of the various crop rotations in meeting the nutritional requirements of the EAT‐Lancet diet (Willett et al. 2019). The optimization process had two objectives: (*i*) fulfilling a dietary energy target (kcal), and (*ii*) minimizing the excesses and deficits in the different food and feed commodities. Furthermore, the algorithm answered to two additional constraints, namely (*iii*) satisfying the eating patterns proposed by the EAT‐Lancet Commission, that is, staying in the upper and lower boundaries of each commodity, as well as (*iv*) achieving solution in the contexts of omnivorous, ovo‐lacto vegetarian or vegan diets. The system boundary is defined at the level of the crop rotation. In this study, tree nuts, fruits and vegetables were considered to be produced externally from the modeled crop rotations, as these are typically produced through horticultural practices and perennial farming. Within this framework, plant‐based components of the diet directly generate “food demand”, whereas animal‐based components translate into a “feed demand”. The annual production of milk, meat, and eggs per functional unit for each livestock species as well as animal feed requirements per category were computed from Wilkinson (2011). For each crop rotation, main production and by‐products levels were specified and constituted the “supply”. Productivities of the different crop species were considered specifically for Wallonia using 5‐year averages of reference statistics (Statbel 2024). In the optimization process, the ranges specified by the EAT‐Lancet diet served as *prior* information, defining a potential range for each commodity. The number of people fed per hectare was treated as a *prior* parameter, to scale the demand according to rotation length. The optimization algorithm then randomly samples values within these *prior* distributions and assesses the capacity of the crop rotation (the supply) to meet the corresponding demand. For each sampled set, an objective function composed of two components was used: (*i*) the gap between the energy supplied and the target intake (to which a higher weight was assigned in the objective function), and (*ii*) the degree of balance between supply and demand across the different food and feed categories. When a crop rotation fails to produce enough of a given food or feed category, the algorithm assumes that the deficit must be covered through imports, but only at the level required to meet the constraints. Conversely, any surplus production within a rotation is treated as excess. This procedure was repeated iteratively throughout the optimization, until the model identified the optimal configuration—namely, the most effective way each crop rotation can contribute to supply the diet pattern.

This decision‐making model enabled the identification of the most efficient use of crop products, byproducts and crop residues, while also allowing for a comparative evaluation across several criteria—such as the number of people fed, the caloric output from crops used for food or feed, the external caloric inputs required, and the livestock population supported by the rotation, expressed in livestock units.

**FIGURE 1:**
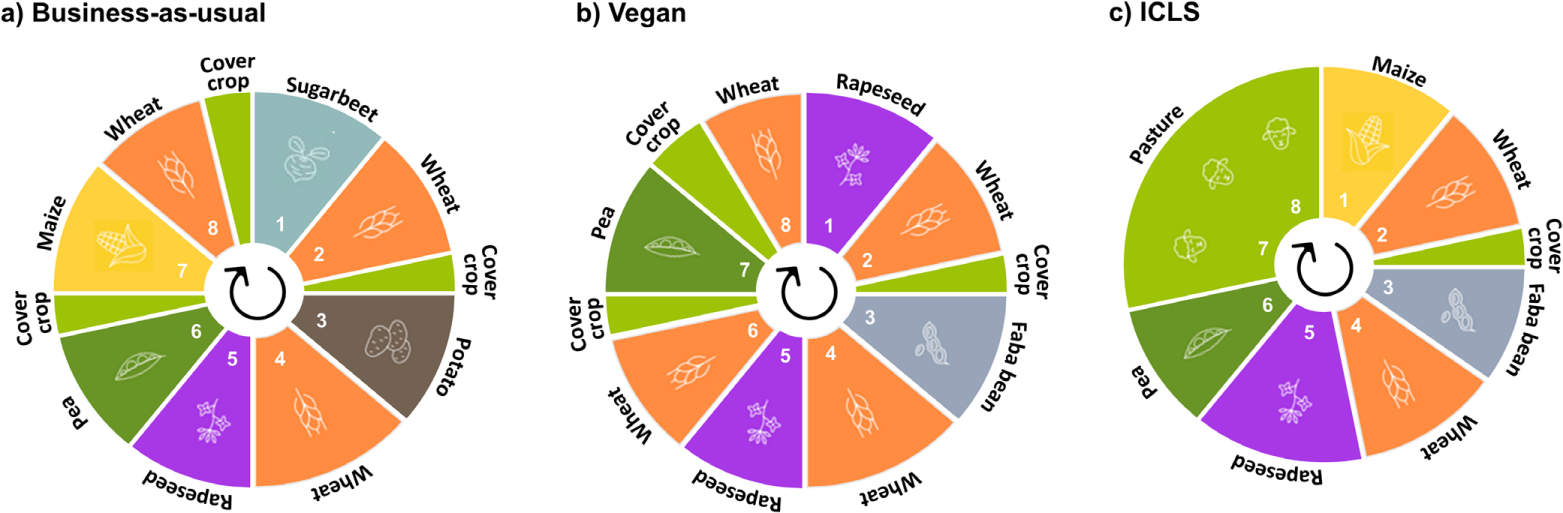
Circularity scenarios, varying in the composition of their crop rotations, their use of crop residues and their integration with livestock. (a) Business‐as‐usual (BAU), reflecting actual Walloon land use and favoring export‐oriented cash crops, with straw‐manure exchange; (b) Vegan, excluding feed crops with no manure and all crop residues incorporated into the soil after harvest; (c) Integrated crop‐livestock system (ICLS), with temporary pastures harvested to feed sheep.

From the outputs of this optimization process, we selected three circularity scenarios differing in (*i*) the types of crops included, (*ii*) the approach to integrating livestock, and (*iii*) the intended dietary composition they aim to supply. Each scenario represents a crop rotation that supports a diet aligned with the nutritional requirements of the EAT‐Lancet diet. However, because these requirements are defined by both upper and lower bounds—allowing flexibility in the consumption of dairy, meat, and other products—each scenario meets them in a different way and under different constraints. These constraints result in varying crop compositions and crop residue management strategies, providing an opportunity to compare the potential outcomes of contrasting choices related to human diets in diversified pedoclimatic contexts in Wallonia. They all last 8 years, which is longer than the usual length of 3–5 years in Wallonia (Statbel 2024), in order to align with good agricultural practices, such as alternating winter and spring crops, while also complying with legal requirements, including the implementation of cover crops before spring planting. As the objective of the study is to conduct systemic rather than factorial comparisons, these three crop rotations substantially differ in their crop compositions, arising from the optimization process explained above. The only constraint that we set to select our three scenarios is to have two simultaneous winter wheat cropping seasons. It allows a granular comparison of their resistance to extreme climatic events, as explained later in the manuscript. Moreover, the technical management specific to each crop (mechanization, tillage and mineral nitrogen fertilization) was identical in all three scenarios and followed common practices in Wallonia, which include stubble breaking (10 cm deep) after cereal harvests and tillage (25 cm deep) for cover crop destruction and after maize harvest and manure application. These crop rotations are described in Figure 1 and, with more details about crop management, in Figures S1–S3.

The *Business‐as‐usual* (BAU) system features the main crops of the loamy Hesbaye region, primarily export‐oriented ‘cash crops’ chosen for their economic value (e.g., potato and sugarbeet). This crop rotation, though longer than typical rotations in Wallonia, was designed to reflect actual Walloon land use within a single rotation cycle incorporating the aforementioned good agricultural practices. Consequently, the proportion of each crop in the rotation closely mirrors their actual share in regional land use (Statbel 2024). It also reflects the current situation where food and agricultural systems are open to heavy imports and exports of commodities. From the perspective of providing a healthy diet, De Clerck et al. (2026) found that this rotation leads to larger deficits in commodities such as oils for food and feed, small deficits in legume crops, and very large surpluses—particularly in potatoes for food and sweeteners derived from sugarbeet (Figure S4). In this system, livestock and cropping operations are separate. Specialized crop farms, like those in the BAU system, import manure from livestock farms in exchange for wheat straw—a common practice in the Belgian Hesbaye region (Figure 1a and S1). Residues for other crops than wheat are incorporated into the soil.

The *Vegan* system aims at reflecting a scenario where livestock would be banished. It therefore focuses on crops intended primarily for human consumption, excluding any feed crops. Within the limitations of a vegan diet, the optimization model described above increased the proportion of cereals to the maximum allowed by the EAT‐Lancet diet in order to make up for the lack of energy‐rich animal‐based foods, while also incorporating rapeseed as an important source of oil. No manure is applied. This system still produces surpluses of feed (e.g., cereal by‐products, oil meals, and other by‐products) (Figure S4). For this reason, all crop residues are incorporated into the soil after harvest (Figure 1b and Figure S2).

The *Integrated Crop‐Livestock System* (ICLS) includes 2.5 years of temporary pastures within an 8‐year rotation (Figure 1c and Figure S3). These pastures are managed as hay meadows, mowed twice a year, with the harvested biomass used to feed sheep. This reflects the most common approach to managing temporary pastures in Wallonia, where mowing is the predominant practice. Similarly, the biomass from cover crops grown within the crop rotation is also harvested and used as sheep feed. As such, we do not simulate direct grazing by sheep on the fields. Instead, we model an ICLS in which there is an exchange of resources: harvested grass and cover crop biomass are exported to feed the sheep, while manure is imported back to the cropping system and sheep live weight gain considered as a production of the system. Sheep are considered to be fed only from this biomass with no supplemental feed imports, since we assume that the 8‐year rotation is continuously present on the farm, as farmers typically cultivate different crops from the rotation simultaneously across different field plots. Although still relatively uncommon, this type of association between crop and sheep farmers is the most prevalent form of ICLS involving sheep in Wallonia. It facilitates farm management while offering agronomic benefits to the crop farm—such as pasture integration and manure use—as well as economic diversification through sheep meat production. The optimization model described above found that the ICLS rotation minimized both imports and exports of food and feed commodities, which remained negligible while provisioning a healthy diet to the local population considering an omnivorous diet (Figure S4). In the first wheat cropping season, wheat straw is removed after harvest for sheep bedding and farmyard manure, while in the second wheat cropping season, it is incorporated into the soil. Further details about ICLS are in Appendix C.

### 2.2 Soil‐Crop Model Simulations

In our study, each scenario is simulated over 24 years—three successive 8‐year rotations—to cover the whole Southern region of Wallonia, which roughly represents half of Belgium. This region is characterized by a temperate climate, with moderate temperatures (average annual temperature of 11°C), heavy cloud cover, and frequent but light rainfall (average annual rainfall of 837 mm). Further details about spatial distribution and seasonal dynamics of temperatures and rainfall are in Figure S5. Its topography and soil composition are highly diverse, spanning the rich, fertile plains of the Hesbaye region, the rugged landscapes of the Condroz region with its plateaus carved by valleys, rivers, and depressions, and the limestone terrains of the Famenne region.

Daily weather data include maximum and minimum temperatures, precipitation, downward surface shortwave radiation, and maximum and minimum relative humidities. For the period 1980–2010, data originate from the reanalysis ERA5. For future climatic conditions, we used three different Earth System Models (ESMs) from the CMIP6 database: MPI‐ESM1‐2‐HR, CMCC‐CM2‐SR5 and MIROC6, respectively denoted MPI, CMCC and MIR in this study. These three ESMs were selected by Sobolowski et al. (2023) as recommended by the CMIP6 forcing for the EURO‐CORDEX exercise. Using the regional climate model MAR (version v3.14; Grailet et al. 2025) forced every 6 h by their atmospheric fields at its lateral boundaries, the three ESMs were downscaled to a 5 km spatial resolution for the period 1980–2100. Each ESM under two SSP scenarios (SSP370 and SSP585) was used to create daily weather data under three global warming rates over 30‐year periods: +2°C (CO_2_: 475 ppm), +3°C (CO_2_: 627 ppm) and +4°C (CO_2_: 1006 ppm), expressing the augmentation of average yearly air temperature at a global scale, compared to the reference period 1850–1900. Future climate scenarios generally predict drier summers, wetter springs and winters, and more frequent extreme rainfall events, with annual rainfalls which do not substantially change. These different climate scenarios allow to compare contrasting climatic conditions (temperatures and pluviometry) under CO_2_ atmospheric concentrations which are similar within each warming scenario between MPI, CMCC and MIR simulations (Table S6), which is interesting to investigate the stability of our circularity scenarios and their impacts on the resistance to extreme climatic events. Further details about climate scenarios are in Appendix D.

Soil parameters originate from SoilGrids maps of soil properties (Poggio et al. 2021), with pedotransfer functions from the R package *euptf2* (Szabó et al. 2021). The spatial resolution is a 250‐m‐sided square (6.25 ha). SOC data were retrieved from Walloon Public Service database, and were provided as averages for the period 2015–2019, with a spatial resolution of 90 m (Vincent et al. 2019; Figure S6), and CaCO_3_ data from the European Soil Data Centre (Orgiazzi et al. 2018). Based on current land use data, only pixels where crops—and not permanent pastures—are cultivated were selected. To reduce the number of simulations, the original dataset of 86,688 pixels—each representing 250‐m‐sided square—was clustered into a smaller set of 11,515 pixels, using unsupervised clustering with the X‐means method (Pelleg and Moore 2000). This method was used across all soils within each 5‐km square climate tile. It was applied based on the full set of soil input data for the model and across all soil horizons. The variables were not considered as covariates in the clustering process. This methodology ensures that even adjacent soil units are not grouped together unless they exhibit strong similarity throughout the entire soil profile and across all variables considered by the model. The soil parameters for these cluster pixels were calculated as the averages of the parameters of the various soils comprising the cluster. Model simulations were then performed at these 11,515 locations, each assigned a unique identifier (UID).

All three circularity scenarios (BAU, Vegan, and ICLS) were simulated in the same manner across each of the 11,515 locations and under each climate scenario. Since temporary pastures are already present in the current land use throughout the entire territory of Wallonia (Walloon Public Service 2024), and since only pixels where crops are actually cultivated were selected, the implementation of any of these circularity scenarios is considered realistic across all pedoclimatic contexts. The objective was to avoid potential bias in comparing the three scenarios that could arise from conducting simulations at a single site unrepresentative of the entire Walloon region.

Model simulations are performed with the soil‐crop model STICS, which is a process‐based model that simulates plant growth as well as water, C and N fluxes (Beaudoin et al. 2022). Model parameterization originates from Delandmeter et al. (2023) and Delandmeter, Colinet, et al. (2024), which successfully modelled field experiments situated in Gembloux and Lonzée, at the center of Wallonia. These experiments comprised the same crops than used in this study, with similar management, and assessed the model ability to simulate crop biomass, yield and SOC dynamics, capturing the influence of environmental drivers (Delandmeter et al. 2023) and the impact of crop residue retention or removal (Delandmeter, Colinet, et al. 2024). Plant parameters for grass are based on Delandmeter, de Faccio Carvalho, et al. (2024). The STICS model ability to simulate N fate, including nitrate leaching, was validated in close agro‐pedoclimatic conditions (e.g., in Northern France (Yin et al. 2020)).

Within ICLS, following the methodology of Delandmeter, de Faccio Carvalho, et al. (2024), sheep live weight gain is computed from dry matter intake, and from live weight gain, the quantity of sheep meat is determined considering a dressing percentage and its proportion available for retail product. Finally, methane (CH_4_) emissions are derived from dry matter intake. Further details are in Appendix C.

### 2.3 Comparison Indicators

The circularity and climate change scenarios are compared over 24‐year simulation periods based on five key services: SOC sequestration, GHG emissions abatement, nitrate leaching reduction, productivity and the stability and resistance of productivity.

#### 2.3.1 Soil Organic Carbon

Although the STICS model internally simulates carbon, water, nitrate and heat transfers in soil using 1 cm thick elementary layers, it outputs SOC at only two depths: over the biologically active layer (0–30 cm) and over the whole soil profile (0–200 cm) (Beaudoin et al. 2022; Delandmeter, Colinet, et al. 2024). Changes in SOC stock (Mg ha^−1^ year^−1^) are hence determined at two different depths: 0–30 cm and 0–200 cm, as an annual average over the 24‐year period.

#### 2.3.2 Greenhouse Gas Emissions

The GHG budget of each scenario is calculated at the farm scale, based on system boundaries defined by the primary and secondary GHG sources associated with C‐N cycles, following the methodology of Autret et al. (2020). Hence, as in Autret et al. (2020), we do not consider secondary sources due to other inputs such as pesticides and PK fertilizers, which are not simulated by the model, neither tertiary sources such as the production and maintenance of equipment. Each budget includes (*i*) soil CO_2_, calculated as the total difference in SOC stock over the whole soil profile (0–200 cm) and the 24‐year period; (*ii*) direct soil N_2_O emissions simulated by STICS through nitrification and denitrification processes, (*iii*) indirect soil N_2_O emissions, based on IPCC emission factors, calculated as the total of 0.75% of the nitrogen lost through leaching and 0.1% of the nitrogen applied as fertilizer (Autret et al. 2020); (*iv*) CO_2_ from fossil fuels emitted during agricultural management practices: combine harvester was considered to consume 20.5 L of fuel ha^−1^, ploughing 27.6 L of fuel ha^−1^ and surface tillage 5.6 L of fuel ha^−1^. The amount of fuel was then multiplied by the emission factor of 0.81 kg C per liter of fuel consumed and by 3.67 to convert a kg C into a kg CO_2_ (Autret et al. 2020); (*v*) CO_2_ emitted during the N fertilizer (urea ammonium nitrate solution) synthesis, computed as the amount of N fertilizer applied (in kg N) multiplied by 6.17 kg CO_2_‐eq kg^−1^ N (Autret et al. 2020); (*vi*) CH_4_ emissions from sheep enteric fermentation within ICLS, computed from live weight gain (Appendix C); and (*vii*) emissions related to livestock manure storage and spreading, which include CH_4_ emissions resulting from the anaerobic decomposition of manure, occurring both during manure storage and on the field, and N_2_O emissions from the storage of manure, either direct through nitrification and denitrification, and indirect through volatile nitrogen losses. In calculating the farm‐scale GHG budget, all manure‐related emissions are accounted for in the ICLS, which includes sheep on the farm. In contrast, for the BAU system, where ruminants are outside the farm, only CH_4_ emissions from manure spreading are considered—alongside soil CO_2_ and N_2_O emissions indirectly liked to manure, which are already included in the preceding GHG categories. The computation methodology of these emissions is based on IPCC (2006) and detailed in Appendix C.

The three GHGs under consideration (CO_2_, N_2_O and CH_4_) are summed up using the concept of Global Warming Potential (GWP). Yet, the standard GWP, such as one based on a 100‐year time horizon, may fail to fully reflect the significant differences in atmospheric lifetimes among the three GHGs under consideration. Therefore, we also use GWP* (Cain et al. 2019; Smith et al. 2021), which treats an increase in the emission rate of a short‐lived climate pollutant (SLCP), such as CH_4_, as equivalent to a one‐time pulse emission of CO_2_, while also accounting for the long‐term impact of CH_4_ emissions by considering past SLCP emissions. This metric is particularly relevant for ruminant livestock production where herd size remains stable (Del Prado et al. 2021, 2023). All GWP metrics used in this study are provided in Table S3.

#### 2.3.3 Nitrate Leaching

STICS simulates at a daily scale the amount of NO_3_‐N leached at the base of the soil profile (2 m deep). These daily amounts are summed up to compute the yearly quantities of nitrate leaching, and the circularity scenarios are compared based on the yearly amounts of NO_3_‐N leached averaged over a period of 24 years.

#### 2.3.4 Productivity

Following Delandmeter, de Faccio Carvalho, et al. (2024), the overall output of each agricultural management approach is evaluated using three distinct measures: (*i*) the total production of grain and pasture biomass (Mg ha^−1^), (*ii*) the energy potential (cereal units; CU ha^−1^) derived from the metabolizable energy content of harvested crops (as referenced by Brankatschk and Finkbeiner 2014), and (*iii*) the financial return (€ ha^−1^), which is calculated based on fixed costs and the average market prices of key commodities over the period from 2018 to 2022. All energetic values, costs and prices are available in Tables S1 and S2. They are all considered constant between climate scenarios. The following crop stresses are also investigated, impacting productivity notably through decreased radiation use efficiency: (*i*) thermal stress, when daily average crop temperature is below or above thresholds; (*ii*) stomatal water stress, based on the available water content in the root zone; (*iii*) nitrogen stress, based on the concept of critical nitrogen concentration; and (*iv*) waterlogging, computed through the proportion of the root profile that is under anoxic conditions (Beaudoin et al. 2022).

#### 2.3.5 Stability and Resistance

The stability of overall productivity, summing yields (metric tons), energetic values (cereal units) and economic values (euros) from all crops within each scenario, as well as sheep live weight gain for ICLS, is computed for each UID as the ratio of the mean productivity of a single rotation (8 years) to its standard deviation across the 24‐year period (Isbell et al. 2015):

$$\text{Stability}=\frac{\mu }{\sigma }$$

The stability is then averaged over all 11,515 UIDs in Wallonia. This metric, by simultaneously accounting for both standard deviation and mean productivity, balances between favoring high but unstable productivity (high *μ*, high *σ*) and low but highly stable productivity (low *μ*, low *σ*) (Delandmeter, de Faccio Carvalho, et al. 2024).

Alongside examining long‐term stability, we also explore the resistance of agroecosystems to extreme climatic events. This refers to their short‐term ability to maintain productivity to near‐normal levels during and after sudden climatic occurrences, such as severe drought or excessive rainfall. In our study, this resistance is compared over wheat yields which occur simultaneously in the three circularity scenarios, that is, the 2nd and 4th crops in each rotation (Figure 1). This methodology (*i*) enables the comparison of the resistance of the same crop during the same years across different circularity scenarios, and (*ii*) provides insights into the impacts of these scenarios on the resistance of wheat, the predominant crop in each scenario and in Wallonia, cereals accounting for 43% of croplands in 2023 (Statbel 2024). In order to enable comparisons across climate scenarios and across the whole Walloon territory, wheat yields were standardized relative to the UID and climate scenarios.

To assess resistance, we classify wheat cropping seasons associated with each yield as ‘normal,’ ‘moderately,’ or ‘extremely’ dry or wet, based on the Standardized Precipitation‐Evapotranspiration Index (SPEI) drought index (Isbell et al. 2015). The SPEI, which quantifies drought onset, duration, and severity by analyzing the balance between precipitation and potential evapotranspiration (Vicente‐Serrano et al. 2010), is calculated monthly over the 24‐year period for each UID. This approach captures the influence of temperature on water demand. Then, following Isbell et al. (2015) and Delandmeter, de Faccio Carvalho, et al. (2024), within each UID and under each climate scenario, climatic events are classified as ‘extreme’ if they occur less than once per decade (falling below the q_0.1_ quantile for extreme dry events and above the q_0.9_ quantile for extreme wet events). They are considered ‘moderate’ if they occur between once per decade and once every four years (falling between the q_0.1_ and q_0.25_ quantiles for moderately dry events and between the q_0.75_ and q_0.9_ quantiles for moderately wet events). Events outside these thresholds are categorized as ‘normal.’ Consistent with Delandmeter, de Faccio Carvalho, et al. (2024), we applied the same quantiles calculated over the historical 1980–2010 period for each climate scenario. This approach means that climatic events are classified as ‘moderate’ or ‘extreme’ relative to current climatic conditions, even if such events may become typical under future climate scenarios. Similarly, to compute the SPEI‐3 index for future climate scenarios, we used the same distribution coefficients derived from the historical 1980–2010 period. Then, for each UID and under each climate scenario, all wheat yields are matched to the SPEI‐3 index associated with the period covering March to May, allowing each growing season to be characterized based on the water balance during the critical 3‐month period preceding flowering (Lenoir et al. 2024). Finally, following Isbell et al. (2015), resistance is calculated as:

$$\Omega =\frac{1}{|\;Y_{e}-\bar{Y_{n}}\;|}$$

where within each UID, $\bar{Y_{n}}$ is the mean productivity during normal years and $Y_{e}$ the productivity during an extreme event (moderately or extremely wet or dry). We favored this definition of the resistance over the original one of Isbell et al. (2015) expressed as $\Omega_{2}=\frac{\bar{Y_{n}}}{|\;Y_{e}-\bar{Y_{n}}\;|}$, to isolate the variability component ($|Y_{e}-\bar{Y_{n}}|$) from the average productivity under normal climatic conditions ($\bar{Y_{n}}$), which can both drive $\Omega_{2}$. $\bar{Y_{n}}$ was therefore investigated separately in order to clearly understand the behavior of both components.

*Multi‐criteria comparison*. The overall performance of the three circularity scenarios across all climatic scenarios is presented in a single figure (Figure 5), comparing side by side their ability to minimize GHG emissions and nitrate leaching, and to maximize soil carbon sequestration, the productivity, and its stability and its resistance to extreme climatic events. The performance for each criterion, calculated using the methodology specific to that criterion as described above, is first averaged across all climatic scenarios. Next, the performance of each circularity scenario is normalized by dividing it by the performance of the BAU scenario, setting the BAU performance to 100. For SOC, however, since the BAU system leads to a reduction in SOC (across the entire soil profile) while the Vegan and ICLS systems increase it, the reference point is adjusted to BAU = −100. In this case, a negative value indicates a further decrease in SOC, while a positive value reflects an increase.

### 2.4 Statistics

Statistical analyses were conducted using the R programming language (R Core Team 2022). Since the data did not meet the normality assumption, group differences were assessed with Dunn's test following a Kruskal‐Wallis analysis. Given the large dataset, these tests were performed on subsamples, each consisting of 1/100th of the total data points, with similar samples used across different scenarios. Outliers were identified using the interquartile range (IQR) method, defined as data points falling below the first quartile (25th percentile) minus 1.5 times the IQR or above the third quartile (75th percentile) plus 1.5 times the IQR.

## 3 Results

### 3.1 Environmental Outcomes

**FIGURE 2:**
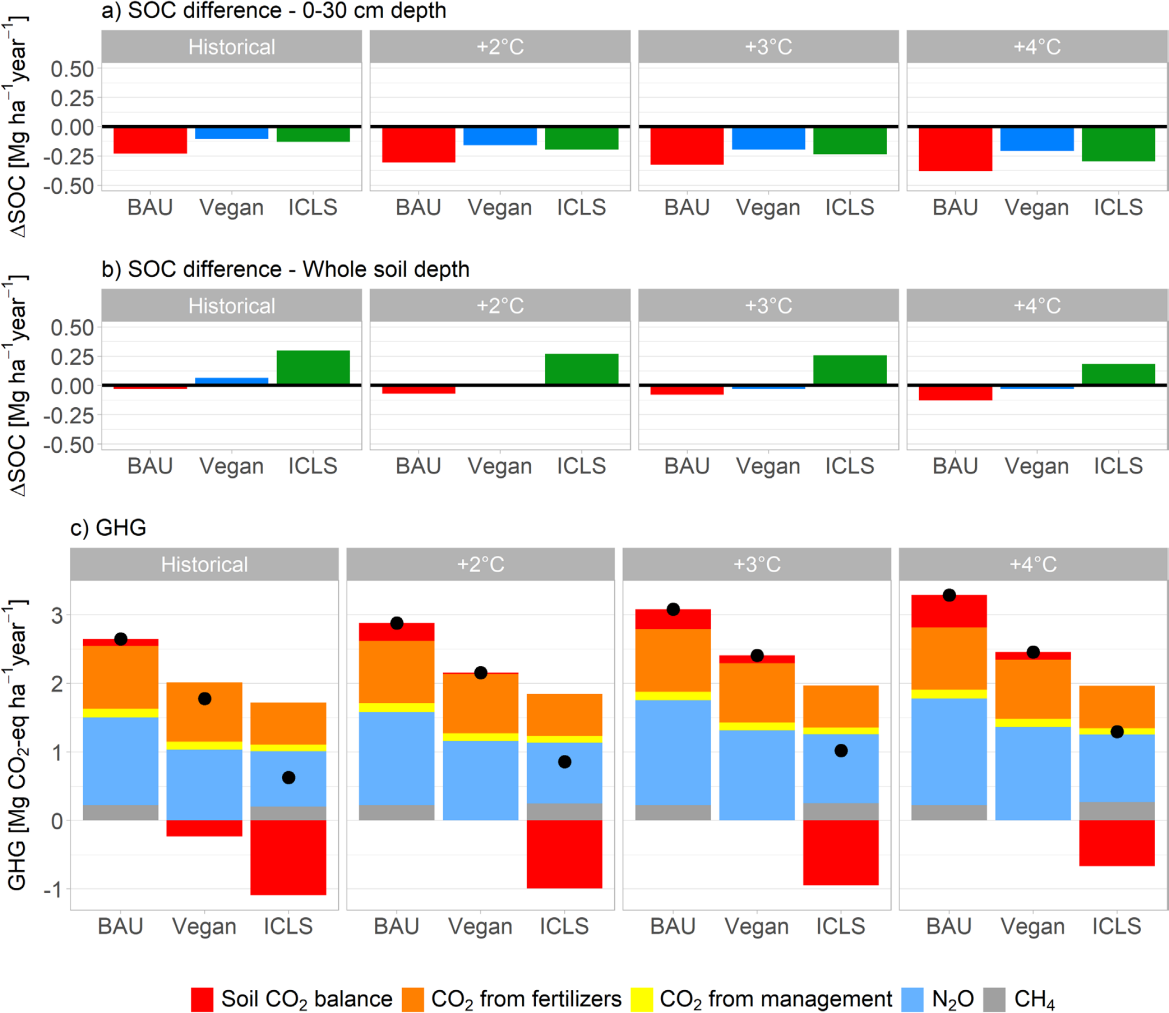
(a) Soil organic carbon (SOC) change (Mg ha^−1^ year^−1^) at 0–30 cm depth; (b) SOC change (Mg ha^−1^ year^−1^) at 0–200 cm depth (whole soil profile); (c) Greenhouse gas (GHG) emissions budget (CO_2_, N_2_O and CH_4_; Mg CO_2_‐eq ha^−1^ year^−1^). CO_2_ from soil = SOC sequestration (negative) or release (positive); CO_2_ from fertilizers = CO_2_ emitted during N fertilizers manufacturing; CO_2_ from management = CO_2_ emitted due to fossil fuel use during agricultural management practices; N_2_O = direct and indirect soil N_2_O emissions, from the field and, within ICLS, also from manure storage; CH_4_ = methane emissions resulting from the decomposition of manure spread on the field, and, within ICLS, also from sheep enteric fermentation and from manure storage (see Methods). Black points show net GHG budget of each scenario (sum of positive and negative emissions contributions). Global warming potential of GHG is computed as GWP* (Smith et al. 2021; Table S3). For all three subplots (a, b and c), SOC change and GHG budget are averaged between Earth System Models (MPI, CMCC and MIR) for climate change scenarios (+2°C, +3°C and +4°C).

All circularity scenarios lead to an average decrease in SOC at 0–30 cm depth (e.g., −0.23 Mg ha^−1^ year^−1^ in average for BAU in 1980–2010, for an average initial SOC stock of 78 Mg ha^−1^ in 0–30 cm in Wallonia; Figure 2a), and future climate change conditions diminish SOC sequestration in both topsoil and subsoil (Figure 2). In all climate scenarios, ICLS reveal to be the only crop rotation increasing SOC over the whole soil profile (up to +0.3 Mg ha^−1^ year^−1^ in average in 1980–2010; Figure 2b).

All circularity scenarios are net GHG emitters, principally due to N_2_O soil emissions and CO_2_ emitted during N fertilizer production (respectively 49% and 35% of total GHG emissions). The intensity of GHG sources, that is, net positive GHG emissions balances, is expected to increase under future climate change scenarios, primarily due to diminished soil carbon sequestration (Figure 2c). Across all climate scenarios, methane emissions have a greater influence when GWP values are used instead of GWP*, though they do not alter the hierarchy among circularity scenarios (Figure S8). Nonetheless, ICLS consistently result in the lowest GHG emissions—30% and 14% lower than BAU and Vegan, respectively, across all climate scenarios—driven primarily by reduced N_2_O emissions (Figure S7) and lower N fertilizer use. This leads to a lower overall GHG source intensity due to the concomitant greater soil carbon sequestration (68% and 57% lower compared to BAU and Vegan, across all climate scenarios). In the opposite, the BAU scenario is the largest GHG source (35% higher compared to Vegan across all climate scenarios), emitting in average 2.6 Mg CO_2_‐eq ha^−1^ in 1980–2010 (Figure 2c).

Nitrate leaching mostly occurs in Southern soils (Figure S9), and averages around 4.5 ± 0.7 kg N ha^−1^ year^−1^ in historical climatic conditions (Figure S10). It slightly increases in climate change scenarios, especially within the Vegan system (up to +193% in +4°C (MPI) scenario compared to 1980–2010). Across all climate scenarios, the Vegan system leads to the highest nitrate leaching levels (57% more and 186% compared to BAU and ICLS, respectively), followed by the BAU system (82% more compared to ICLS) (Figure S11).

### 3.2 Agronomic and Economic Productivity

**FIGURE 3:**
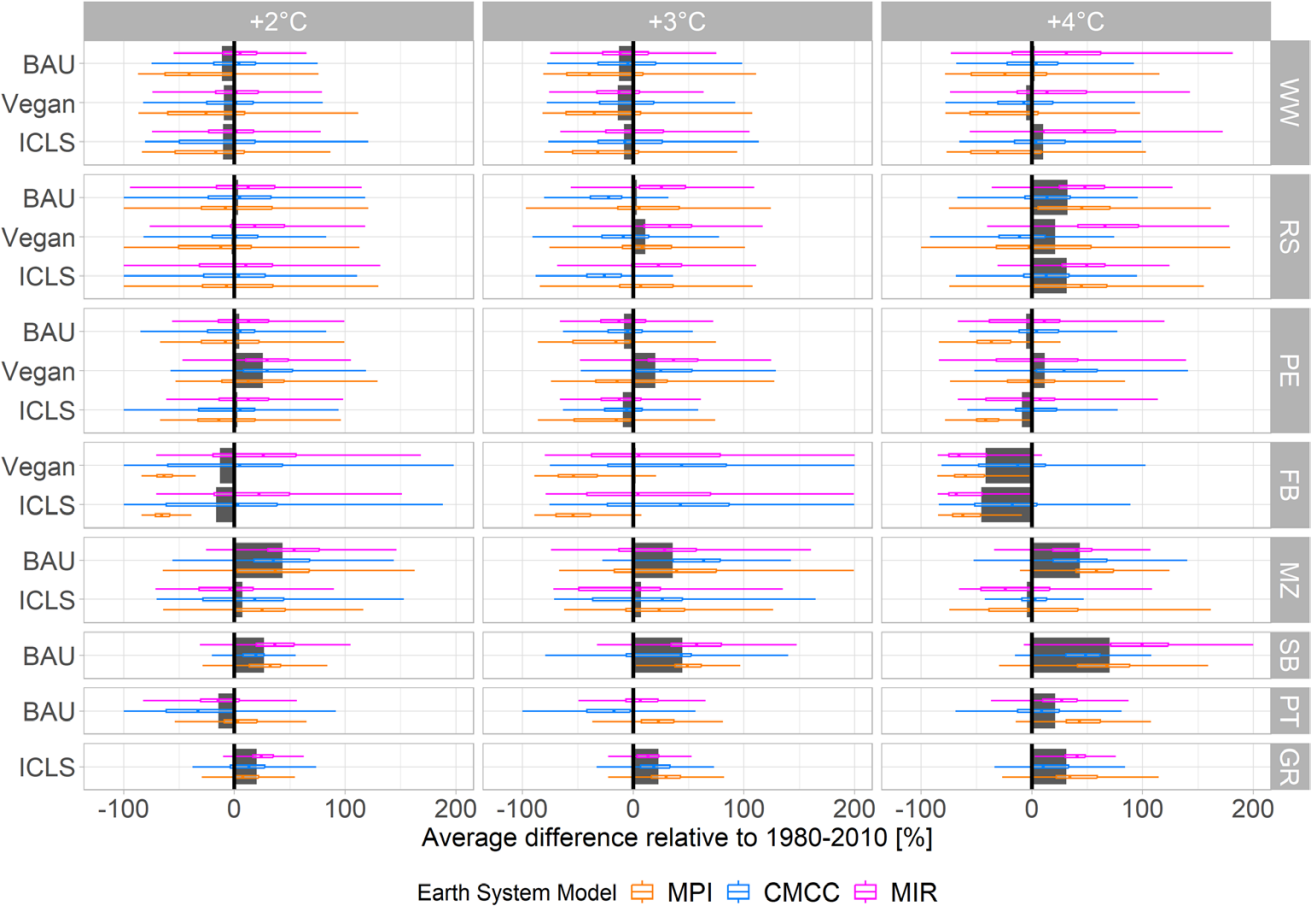
Yield evolution compared to the historical period 1980–2010 for each circularity scenario (left‐hand legend) and each crop (right‐hand legend). Grey bars represent yield evolutions averaged over Earth System Models (MPI, CMCC, MIR). Colorized boxplots represent yield evolutions for each Earth System Model. FB, faba bean; GR, grass; MZ, maize; PE, pea; PT, potato; RS, rapeseed; SB, sugar beet; WW, winter wheat.

In future climate change scenarios, yield evolutions would greatly vary between crops and between climate scenarios (Figure 3). Under +2°C scenarios, most winter crops would suffer average yield losses or very small gains. With CO_2_ elevation associated to higher warming scenarios, compensation might appear, especially for rapeseed which would substantially increase its average productivity (−44% for faba bean, −1% for pea, +3% for wheat and +28% for rapeseed compared to the historical period, in average under +4°C scenarios across all circularity scenarios; Figure 3 and Figure S11). These yield losses are associated with an augmentation in both waterlogging and stomatic water stress (Figure S12). Summer crops such as sugar beet, maize and, to some extent (only in +3°C and +4°C scenarios), potato, would increase their yields, reaching average augmentations up to +70% for sugar beet, +19% for maize and + 21% for potato under +4°C scenarios compared to 1980–2010 (Figure 3 and Figure S11).

The BAU system achieves the highest overall productivity, regardless of the metrics used (e.g., +45% and +82% compared to Vegan and ICLS in 1980–2010 when expressing productivity as energetic value; Figure S13). Moreover, this trend is expected to intensify under future climate change scenarios due to BAU reliance on summer crops (Figure S13). While the Vegan system ranks second in productivity when measured in energetic value (+25% compared to the ICLS in 1980–2010), the ICLS system holds this position when measured in yields and economic value, moreover in future climate scenarios (+37% and +32% compared to Vegan under +4°C scenarios, using respectively yields and economy as metrics; Figure S13).

### 3.3 Stability and Resistance

Expanding model simulations to large spatio‐temporal scales allows for a more comprehensive assessment of productivity stability, expressed as the ratio of the mean productivity of an 8‐year rotation to its standard deviation over the 24‐year period. As crop yield variability increases under future climate scenarios (Figure 3 and S11), productivity stabilities systematically decrease under climate change scenarios (Figure S14). ICLS generally have the largest stabilities compared to the two other systems, moreover when productivity is expressed in economic value (+44% and +86% compared to BAU and Vegan across all climate scenarios), except for yields and energetic productivities in 1980–2010 where it is the BAU system which is the most stable (Figure S14).

**FIGURE 4:**
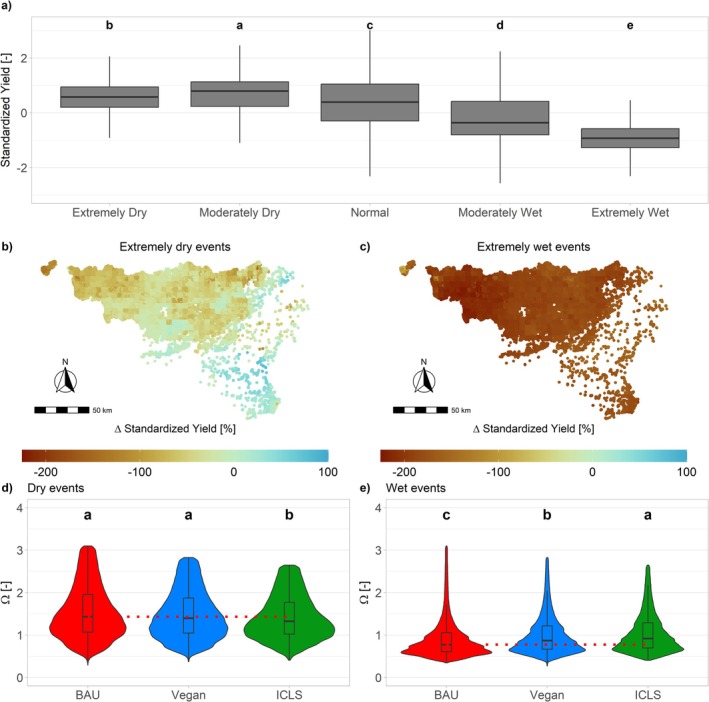
Impacts of extreme climatic events on winter wheat yields across Wallonia (Belgium). (a) Wheat yields across all circularity scenarios, standardized relatively to the UID and climatic scenarios, as function of climatic conditions in March–May determined by the SPEI‐3 drought index. Each map represents the yield difference between normal and extremely dry (b) and wet (c) climatic conditions, across all circularity and climate scenarios (difference being calculated between standardized yields, as computed in (a)—hence potentially reaching values inferior to −100%). For each circularity scenario, boxplots show the resistance (Ω, see Methods) to extremely dry (d) and wet (e) events (outliers not shown). Horizontal lines display medians, and dotted red lines display BAU medians for reference. Violin plots display the distribution of Ω across circularity scenarios, each violin representing a kernel density estimate of the Ω distribution, with the width indicating the probability density. Different letters above the boxplots denote statistically significant differences between groups as determined by Dunn's test (*p* < 0.05).

We also examine how well the rotations can endure a sudden extreme climatic event, a characteristic referred to as *resistance*. This is evaluated by comparing winter wheat yields across different circularity scenarios under *moderate* or *extreme* climatic conditions versus conditions characterized as *normal* within historical climatic conditions (see Methods). First, results show that *normal* climatic conditions will become increasingly rare in the future (Figure S15). Under such conditions, the BAU system leads to slightly higher wheat yields than Vegan and ICLS (Figure S16). In contrast to this declining occurrence of *normal* climatic conditions, *extreme*, and to a lesser extent, *moderate* wet events will increase in frequency (Figure S15), and significantly reduce wheat yields and increase their variability (Figure 4a,c). ICLS demonstrates slightly greater resistance to these events compared to other circularity scenarios (+14% and + 4% compared to BAU and Vegan; Figure 4e), followed by Vegan (+10% compared to BAU). Dry—and often sunnier—periods appear to have a contrasted impact on wheat productivity, leading to either slight gains or losses in yield (Figure 4a,b). Therefore, increasing resistance to dry events is not crucial, and the Vegan and ICLS systems exhibit the lowest resistance to such conditions (Figure 4d).

**FIGURE 5:**
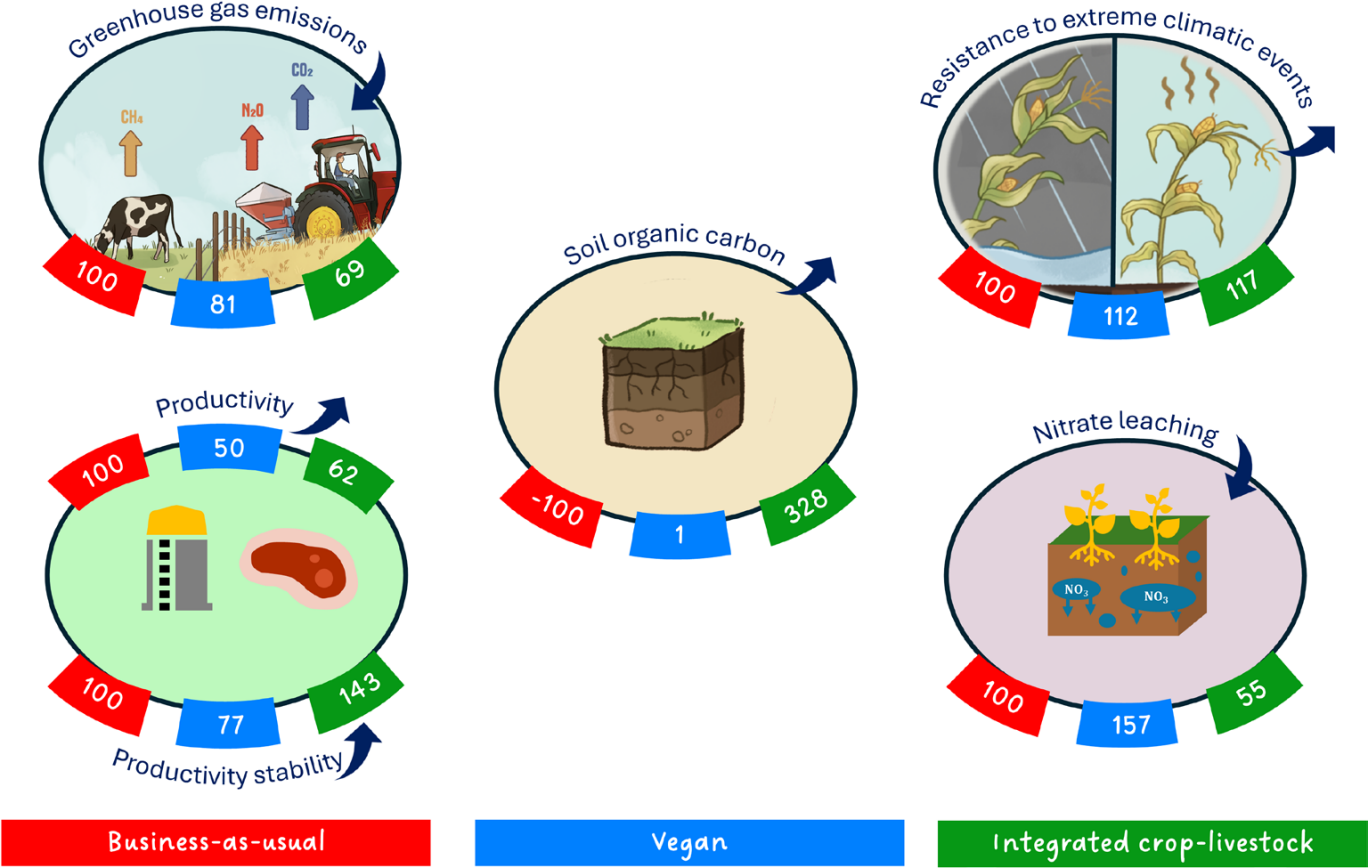
Overview of the five main performance metrics of the three circularity scenarios (Business‐as‐usual (BAU), Vegan, Integrated crop‐livestock (ICLS)). Each category includes an arrow indicating whether the criterion is intended to be maximized (upward arrow) or minimized (downward arrow). Values are dimensionless averages across all climate scenarios, using BAU as the reference point (BAU = 100; see Methods for computation details). For soil organic carbon (SOC), since the BAU system reduces SOC (over the whole soil profile) while Vegan and ICLS increase it, the reference point is set at BAU = −100. The resistance values (upper right of the figure) represent the average resistance to moderately and extremely wet events, which have the greatest impact on wheat yields (see Methods and Figure 4).

The main impacts of the circularity scenarios on SOC, GHG emissions, nitrate leaching, productivity and its stability and resistance to extreme climatic events are summarized in Figure 5.

## 4 Discussion

Three different agronomic scenarios—business‐as‐usual, vegan and integrated crop‐livestock systems—were investigated, varying in the composition of their crop rotations, their use of crop residues and their integration with livestock. They were compared over 30‐year periods and over the whole Walloon region of Belgium, covering 541,800 ha, under +2°C, +3°C and +4°C global warming scenarios and using three different climate models. From soil‐crop modelling outputs, we evaluated their multi‐criteria performance in relation to provisioning, supporting, and regulating ecosystem services.

Reinforcing the model validation discussed in the Material and Methods, our model simulations under historical climatic conditions prove to be in accordance with observed trends in Wallonia for yields (Table S4), SOC, nitrate leaching and nitrous oxide emissions (Table S5).

### 4.1 The Evolution of Productivity Under Climate Change

Climate change has a significant impact on productivity in Wallonia, with effects varying by crop type. Summer crops, particularly C_3_ crops, generally benefit from the CO_2_ fertilization effect (Toreti et al. 2020) and rarely experience water shortages (Figure S12). In contrast, winter crops are negatively affected, suffering from waterlogging and exhibiting high yield variability (Gobin 2010). Yet climate change impacts on crops heavily rely on crop and ESMs used (Wang et al. 2024) and whether cropping periods are adapted in response to changing weather patterns, as was not the case in this study (Vanuytrecht et al. 2016). This study primarily aims to compare circularity scenarios within the context of climate change, emphasizing the importance of crop rotation design and crop selection based on their potential future yield evolution. Rather than providing absolute estimates of climate change impacts on crops in Belgium—an analysis better suited to multi‐model ensembles (Wallach et al. 2016)—this study focuses on relative comparisons.

### 4.2 Stability and Resistance to Extreme Climatic Events

Given the observed increased variability in crop yields under climate change, we leveraged various ESMs across different warming scenarios to capture a broad spectrum of climatic conditions. This enabled a detailed analysis of agroecosystem stability and resistance to extreme weather events. Our results highlight that ICLS represent the optimal management scenario for enhancing stability, notably due to its diversified production including pastures—found to be relatively more stable than crops (Figure 3)—and sheep meat, and increasing resistance to extreme wet events (Szymczak et al. 2020; Sekaran et al. 2021). These extreme climatic events have been shown to have, in Belgian pedoclimatic contexts, the greatest impact on productivity (Figure 4) and are projected to increase in frequency under future climate scenarios (Figure S15; Mbow et al. 2019). ICLS pastures contribute to increased SOC (Franzluebbers and Martin 2022), thereby improving water retention and climate resilience (Lal 2020). This is especially crucial as climate change is shown to reduce SOC (Figure 2a) due to accelerated mineralization from rising temperatures (Crowther et al. 2016).

### 4.3 Carbon and Nitrogen Cycles

Our results also indicate that all three agronomic scenarios are net GHG emitters, as are most agroecosystems when indirect emissions such as arising from the production of agricultural inputs and fuel combustion are also considered (Lehuger et al. 2011). In this study, as in Autret et al. (2020), we calculated the farm‐scale GHG budget while also accounting for emissions from nitrogen fertilizer manufacturing, a highly impactful process (Wood and Cowie 2004; Chataut et al. 2023). In our findings, N_2_O soil emissions and CO_2_ emitted during N fertilizer production are found to be the main pools of GHG emissions, in agreement with Van Stappen et al. (2015). The intensity of GHG sources is expected to increase under future climate change scenarios due to reduced carbon sequestration, due to increased mineralization under higher temperatures (Van Gestel et al. 2018), and heightened nitrous oxide emissions, likely driven by increased anoxic conditions (Barnard et al. 2005). This indicates perilous feedback in which food systems drive climate change, which in turn disrupts agriculture and further intensifies global warming (Yang et al. 2024). The ICLS leads to higher carbon sequestration levels than business‐as‐usual and vegan scenarios, which we found to primarily result from the pasture phase, which is often the case in pasture‐crop rotations as noted by Franzluebbers and Martin (2022). Considering SOC not only in the topsoil (0–30 cm) but over the whole soil depth (Figure 2) is important as pastures notably develop deep root systems that contribute to net carbon inputs upon root decomposition. Indeed, the difference in SOC changes between the topsoil and subsoil arises from the assumption by the STICS model that mineralization below 30 cm is considered as negligible (Beaudoin et al. 2022). At such depths, soil carbon is more stable with lower mineralization rates, making it more resistant to increased temperatures induced by climate change (Jackson et al. 2017).

Combined with its lower dependence on synthetic nitrogen fertilizers, ICLS reveals to have lower overall GHG emissions than the two other scenarios. Moreover, it exhibits significantly lower nitrate leaching levels (Figure S10), in accordance with for example, Lemaire et al. (2014). Hence, while crop diversification sometimes displays a trade‐off between increased N supply but soil C accrual which is limited (Yi et al. 2025), here integrating pastures and livestock within cropping systems reflects a synergy between increased C sequestration and reduced N losses.

In contrast, under historical climatic conditions, the BAU scenario results in the highest levels of nitrate leaching, followed by the Vegan scenario—reflecting the hierarchy of average synthetic nitrogen fertilizer use. However, under future climatic conditions, it is the Vegan scenario that exhibits the highest levels of nitrate leaching (Figure S10). Yet this may partly be due to the use of crops that are more susceptible to failure, such as rapeseed, and to the fact that the model continues to apply fertilization even when the crop fails to establish properly—something farmers would typically avoid in practice. Overall, nitrate leaching levels remain low across all three circularity scenarios (4.5 ± 0.7 kg N ha^−1^ year^−1^ in historical climatic conditions), compared to other experiments in Belgium with amounts of leached nitrate up to 10 times higher, especially when no cover crops are sown following spring crops (De Waele et al. 2017). The relatively low levels of nitrate leaching in our simulations can also be attributed to the long‐term nature of these crop rotations (Beillouin et al. 2021). They are in the range of other field experiments conducted under similar agronomic and pedo‐climatic conditions, involving cover crops and analogous cash crops with comparable fertilization levels (Yin et al. 2020; Plaza‐Bonilla et al. 2015; Becel et al. 2015).

### 4.4 Choices in the Design of Circularity Scenarios

The Vegan scenario, simulating an agriculture where livestock would be banished, limits SOC loss compared to business‐as‐usual (Figure 2a,b). It may primarily result from its emphasis on cereals and rapeseed, as these crops exhibit greater root biomass than others like potato or sugarbeet (Heinemann et al. 2023; Iwama 2008), resulting in increased carbon inputs to the soil. Root‐derived carbon is a key contributor to SOC, as it remains in the soil two to three times longer than carbon from aboveground residues or manure (Poeplau et al. 2021; Kätterer et al. 2011). Moreover, the absence of manure in the Vegan system is compensated by the systematic incorporation of crop residues into the soil, even if crop residues have a lower C‐retention coefficient compared to organic manure (Maillard and Angers 2014). However, the Vegan scenario demonstrates lower and less stable productivity (Figure 5). In contrast, the ICLS leads to lower GHG emissions—even when accounting for livestock and manure methane emissions using two different GWP metrics (Figure 2b and Figure S8). Methane emissions in the BAU scenario are quite similar in magnitude but differ in origin. Rather than arising directly from enteric fermentation, they result from manure spreading. However, a comprehensive life cycle analysis—beyond the scope of this study—might also attribute some enteric fermentation emissions to the BAU scenario, from livestock farms that exchange manure for wheat straw. Additionally, such an analysis could account for emissions associated with infrastructure, pesticide use, and other indirect sources across all scenarios. For example, when aiming to provide a nutritionally adequate diet for the local population, each scenario still results in surpluses and deficits of food and feed commodities. These imbalances affect the capacity of the systems to supply a fully local, self‐sufficient human diet—with the BAU and Vegan systems showing much larger discrepancies than the ICLS (Figure S4). Furthermore, the required imports and exports originate from production systems outside the boundaries of this study, which are linked to additional land use, environmental impacts, and CO_2_ emissions associated with transportation. It is also important to note that the three crop rotations investigated in this study are not unique system modalities aligned with different circularity schemes under contrasting dietary constraints. For example, while this study assumed similar crop management practices across all scenarios—notably to enable a comparison of resistance capacities to extreme climate events—one could envision the Vegan and ICLS scenarios diversifying their nitrogen sources—drawing, for instance, from human excreta, compost, and other alternatives (De Boer and Van Ittersum 2018). We could also investigate ICLS with temporary pastures which would not be mowed—as it is most often the case in Wallonia—but actually grazed. Yet this would require adapting the modelling methodology by performing very frequent cuts in the sward biomass and adapting the return of feces and urine, as in Delandmeter, de Faccio Carvalho, et al. (2024). Finally, it may be worthwhile to explore differentiated land‐use allocation across the various circularity scenarios—for example, prioritizing cash crops on deep, fertile soils and reserving poorer soils for pastures. By simulating food and feed exchanges between these regions using crop model outputs, one could assess the trade‐offs between promoting circularity and hence nutrient cycle closure and soil fertility enhancement, and optimizing land use based on the specific characteristics of each pedoclimatic region.

### 4.5 The Importance of Multi‐Criteria Comparisons

Our findings highlight the importance of considering a broad range of ecosystem services when comparing agronomic scenarios. For instance, while the business‐as‐usual system demonstrates larger productivity, it is also highly variable and contributes to greater environmental impacts, such as increased GHG emissions. In contrast, the crop‐livestock system presents a more balanced trade‐off, with lower but more stable and resistant productivity altogether with reduced environmental impacts. We therefore emphasize the need for a transparent approach to comparative metrics, as their definition can influence outcomes—potentially favoring high but variable productivity over low yet stable productivity, or vice versa.

In conclusion, this study reveals the benefits of crop‐livestock systems in terms of climate change adaptation, through stability and resistance to extreme climate events, and mitigation, through reduced GHG emissions and nitrate leaching. It furthermore demonstrates that crop models, when integrated with innovative methodologies for crop rotation design and resistance assessment, can serve as reference tools for exploring the connection between farm‐level circularity, human diet, and climate change across large spatio‐temporal scales. The necessary transformation of the food system for greater sustainability requires considering the feedback between agriculture and human diet. This highlights the need for further studies to better understand this relationship across diverse cultural and pedoclimatic contexts.

## Author Contributions

**Mathieu Delandmeter:** conceptualization, formal analysis, investigation, methodology, software, visualization, writing – original draft, writing – review and editing. **Bruno Basso:** methodology, supervision, validation, writing – review and editing. **Xavier Fettweis:** data curation, validation, writing – review and editing. **Christophe Lacroix:** data curation, writing – review and editing. **Pierre Aubry:** data curation, writing – review and editing. **Jérôme Bindelle:** conceptualization, methodology, supervision, validation, writing – review and editing. **Benjamin Dumont:** conceptualization, methodology, supervision, validation, writing – review and editing.

## Funding

This work was supported by Fonds De La Recherche Scientifique (FNRS, 44221, 549 R.8003.20).

## Conflicts of Interest

The authors declare no conflicts of interest.

## Supporting information


**Data S1:** Supporting Information.

## Data Availability

The data that support the findings of this study are openly available in https://doi.org/10.6084/m9.figshare.31293703.
