# Supplementary material for: Livestock Integration Into Cropping Systems Enhances Their Climate Change Resistance and Mitigation While Reducing Their Environmental Impacts
Source: Glob Chang Biol. 2026 Feb 25;32(2):e70765. doi: 10.1111/gcb.70765 (PMC12936454; doi:10.1111/gcb.70765)
Supplement: Supplementary file 1 — Data S1: Supporting Information. [file GCB-32-e70765-s001.pdf]

Livestock integration into cropping systems enhances their climate change adaptation and reduces their environmental impacts

Mathieu Delandmeter, Bruno Basso, Xavier Fettweis, Christophe Lacroix, Pierre Aubry, Jérôme Bindelle, Benjamin Dumont

SUPPLEMENTARY MATERIAL

Table of Contents

A. Supplementary Figures 2
B. Supplementary Tables 18
C. Livestock and manure simulation 23
C.1. ICLS simulation 23
C.2. GHG budget computation linked to manure 24
D. Weather data 26
E. STICS inputs and outputs data 26
References for Supplementary Material 27

A. Supplementary Figures

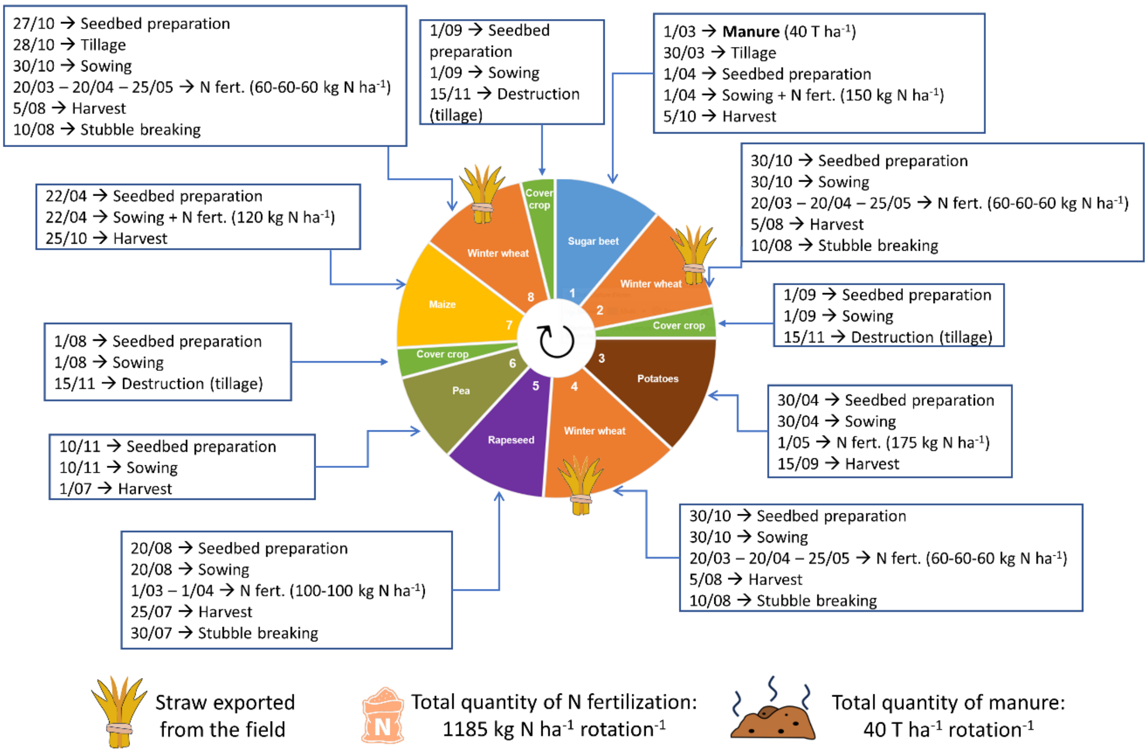

Figure S1. Crop management for the *Business-as-usual* (BAU) system. Stubble breaking = 10 cm deep, tillage = 25 cm deep, seedbed preparation = 7 cm deep.

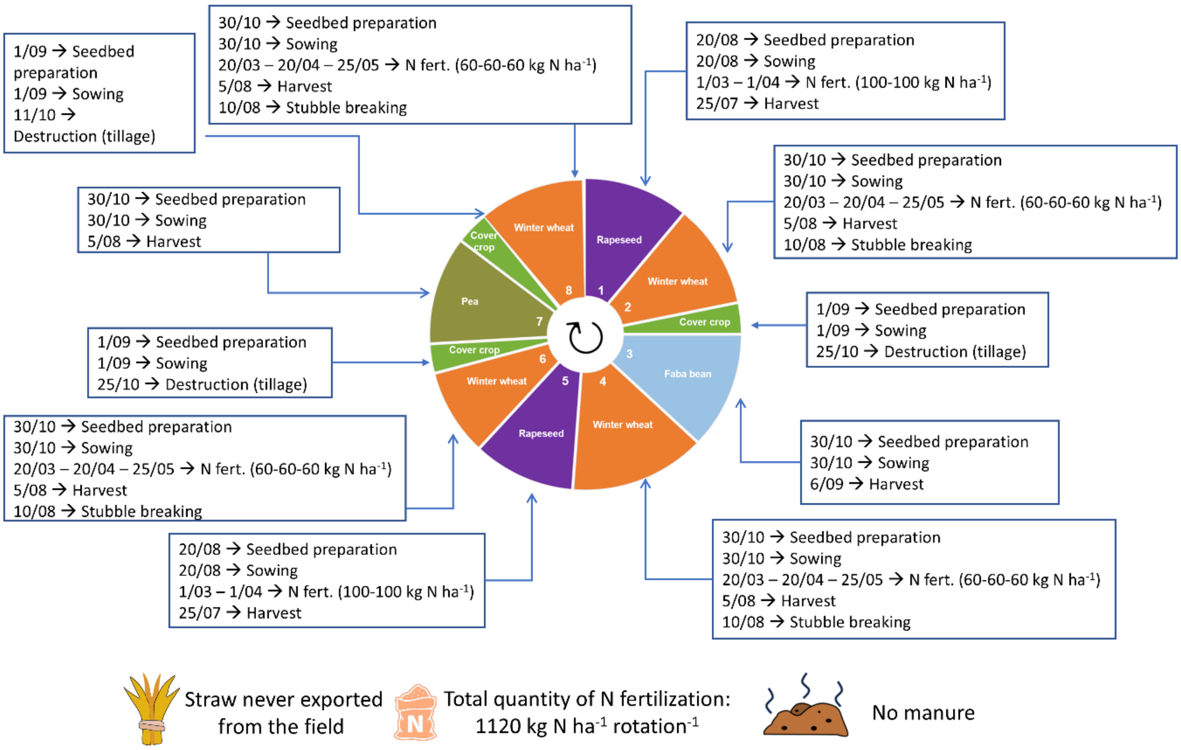

Figure S2. Crop management for the *Vegan* system. Stubble breaking = 10 cm deep, tillage = 25 cm deep, seedbed preparation = 7 cm deep.

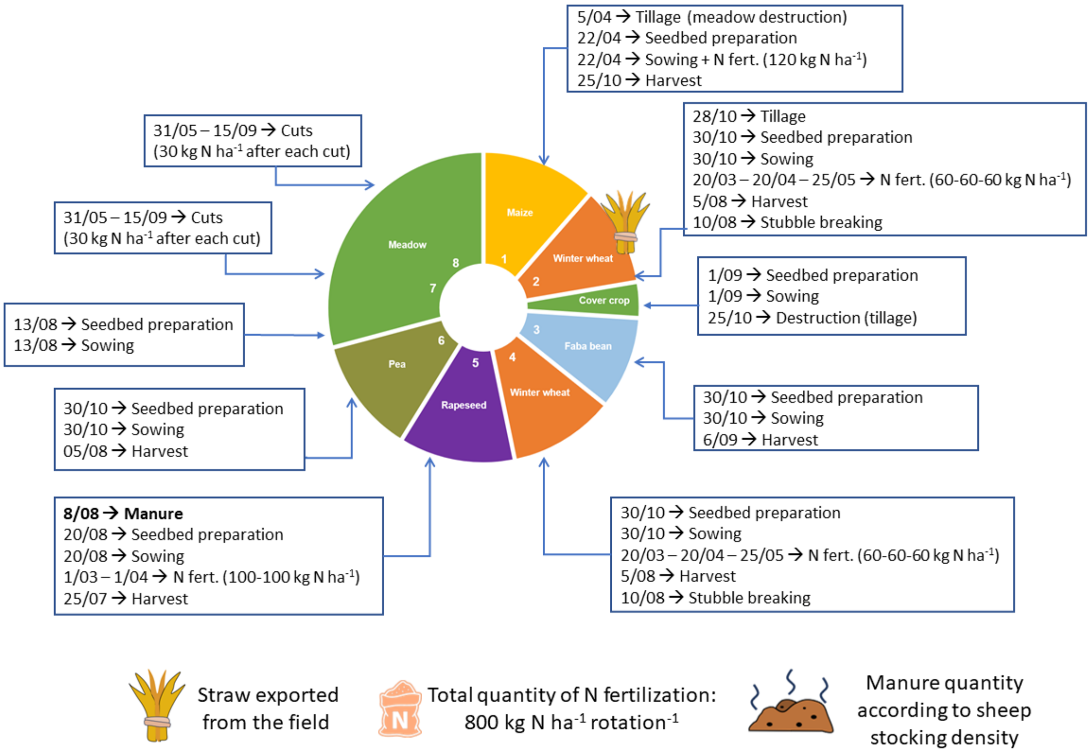

Figure S3. Crop management for the *Integrated crop-livestock system* (ICLS). Stubble breaking = 10 cm deep, tillage = 25 cm deep, seedbed preparation = 7 cm deep. Manure quantity was computed following the simulation methodology described in Appendix C (Equations B1 and B2).

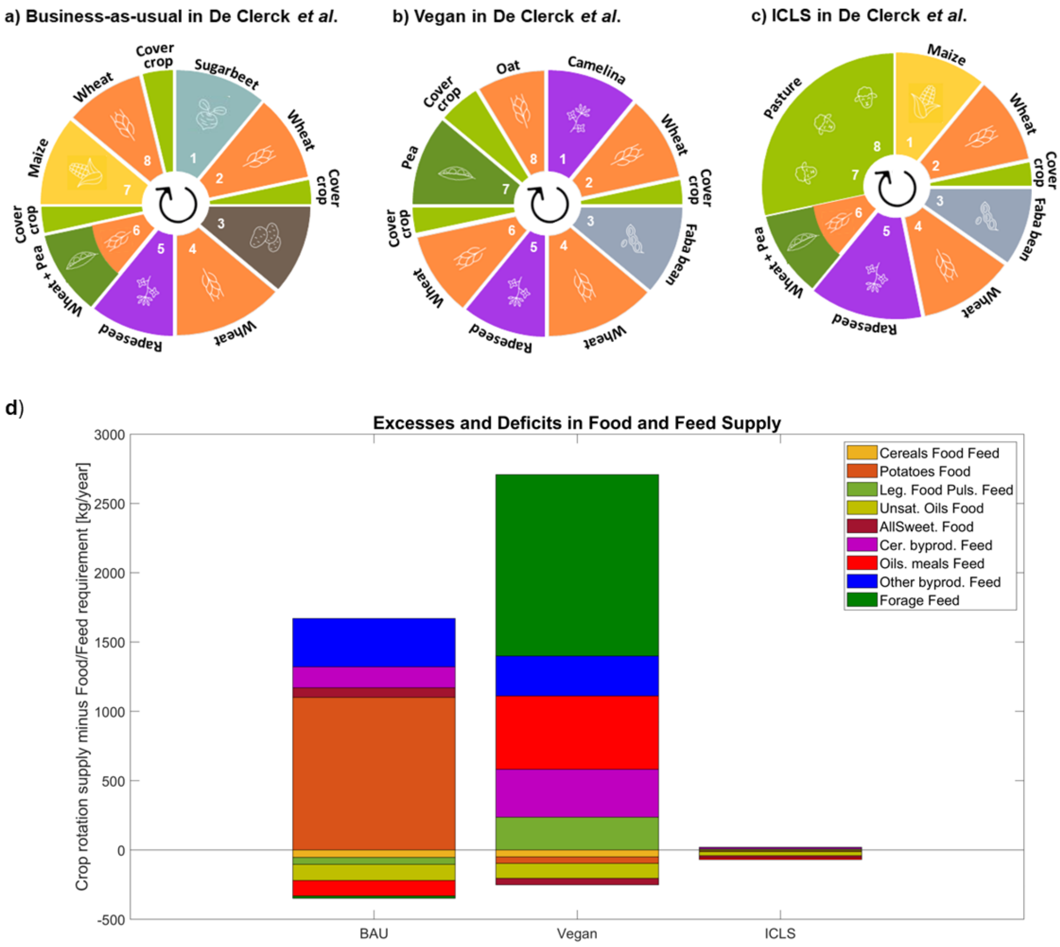

Figure S4. Scenarios selected among the 40 scenarios compared in De Clerck et al. (2025): a) business-usual (BAU); b) Vegan and c) Integrated Crop-Livestock System (ICLS). d) Excesses and deficits in food and feed commodities compared to the EAT Lancet requirements (Willett et al., 2019), for the BAU, Vegan and ICLS scenarios from De Clerck et al. (2025). These excesses and deficits were computed to provide an omnivorous diet for the BAU and ICLS scenarios, and a vegan diet for the Vegan scenario. The Figure A4d is adapted from De Clerck et al. (2025). In the original work by De Clerck et al. (2025), a wheat-pea association was evaluated within the BAU and ICLS rotations. In our study, to ensure valid crop model simulations, this association – which is not validated in the standard version of the STICS model – was replaced by a sole pea crop. Likewise, for the same reasons, in the Vegan system, camelina cultivation was replaced with rapeseed, and oat was replaced with winter wheat.

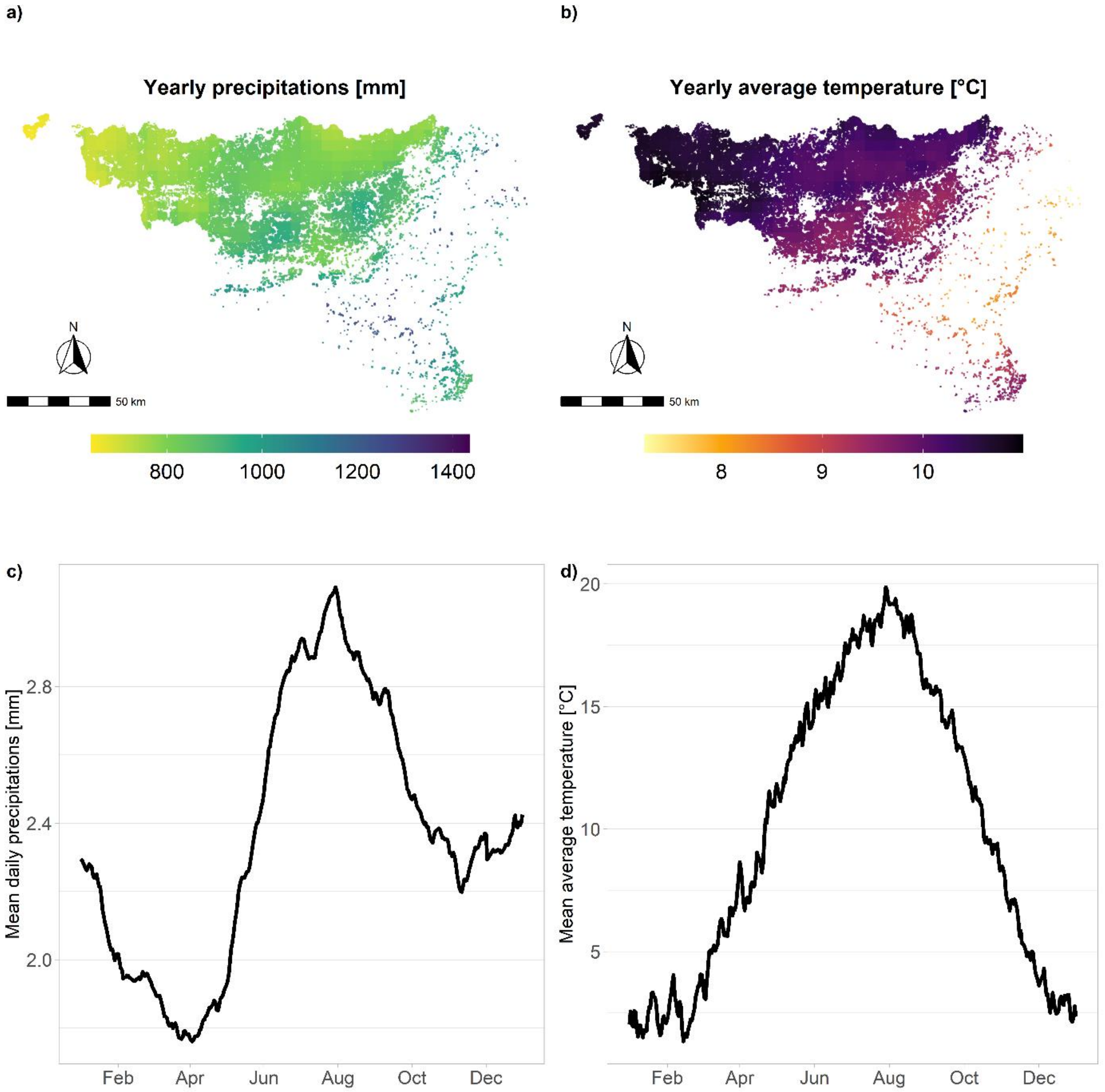

Figure S5. Climate characteristics in Wallonia for the historical climatic conditions (1980-2010). a) yearly quantity of precipitations, b) yearly average temperature, c) dynamics of the daily precipitations over a year, averaged over the Walloon region and over the 30-year period, and d) dynamics of the temperature, averaged over the Walloon region and over the 30-year period. For Fig S5c, the dynamics of precipitations were smoothed out by averaging the daily precipitations over a sliding window of 60 days.

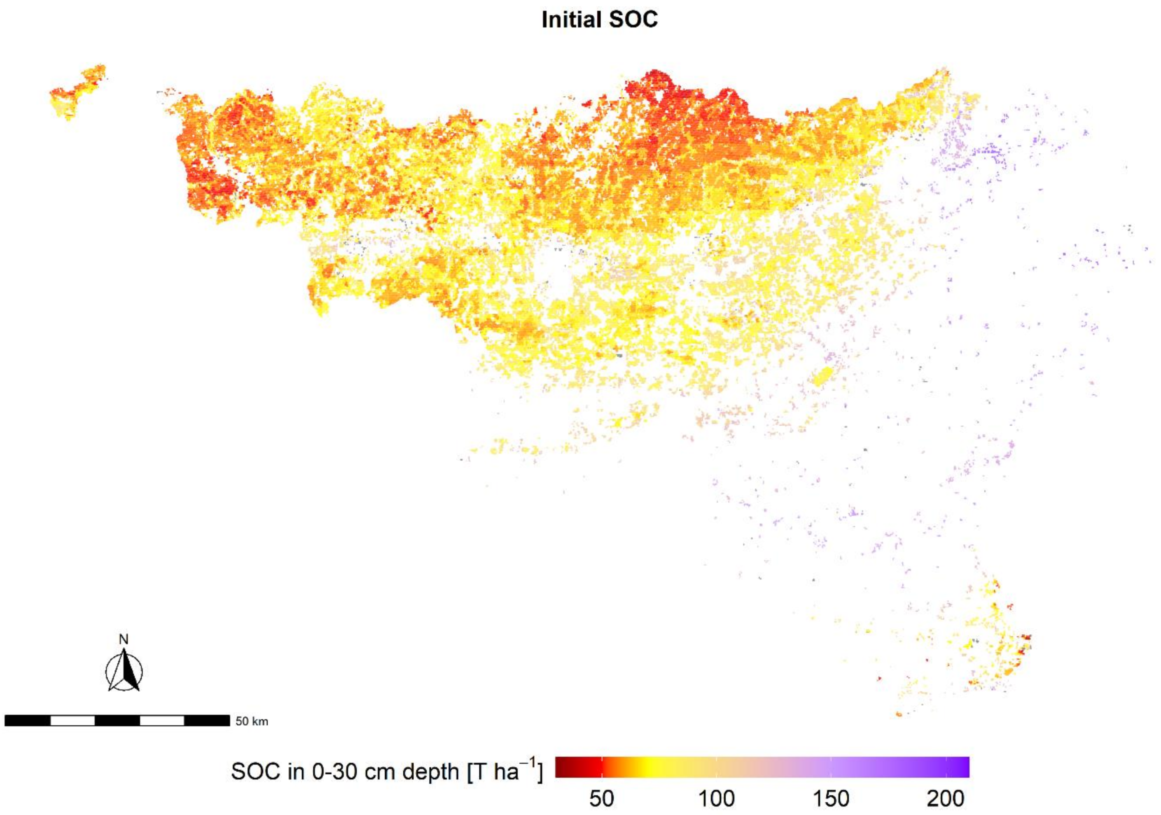

Figure S6. Initial soil organic carbon (SOC) stock in 0-30 cm depth, similar for all circularity and climate scenarios.

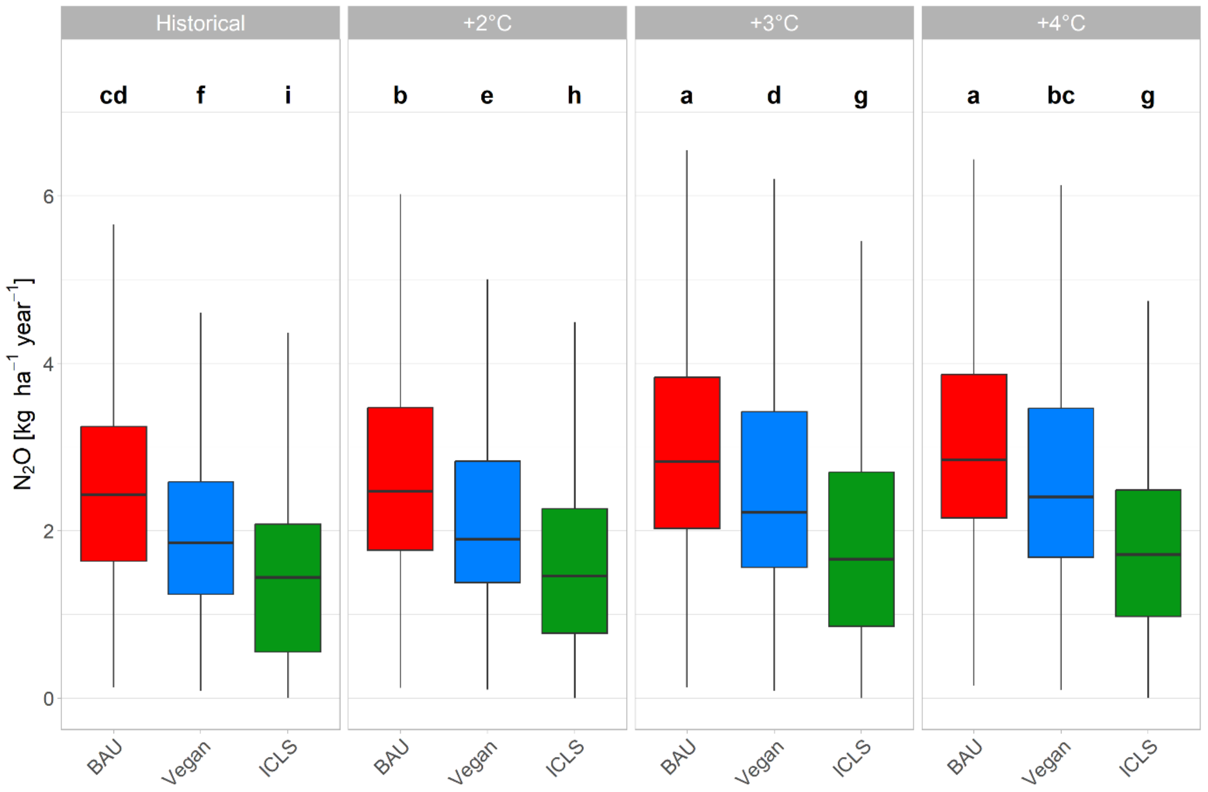

Figure S7. Direct nitrous oxide emissions (N_2O-N; kg ha^{-1} year^{-1}) from the soil (nitrification and denitrification) for the different circularity (BAU, Vegan, ICLS) and climate (historical, +2°C, +3°C, +4°C) scenarios, averaged over the 24-year simulation period across Wallonia. For climate change scenarios (+2°C, +3°C and +4°C), N_2O emissions are averaged between Earth System Models (MPI, CMCC and MIR). Boxplots show the interquartile range (25th – 75th percentiles), with medians displayed by horizontal lines and minimal and maximal values displayed by vertical lines (outliers not shown). Different letters above the boxplots denote statistically significant differences between groups as determined by Dunn's test (*$p < 0.05$*).

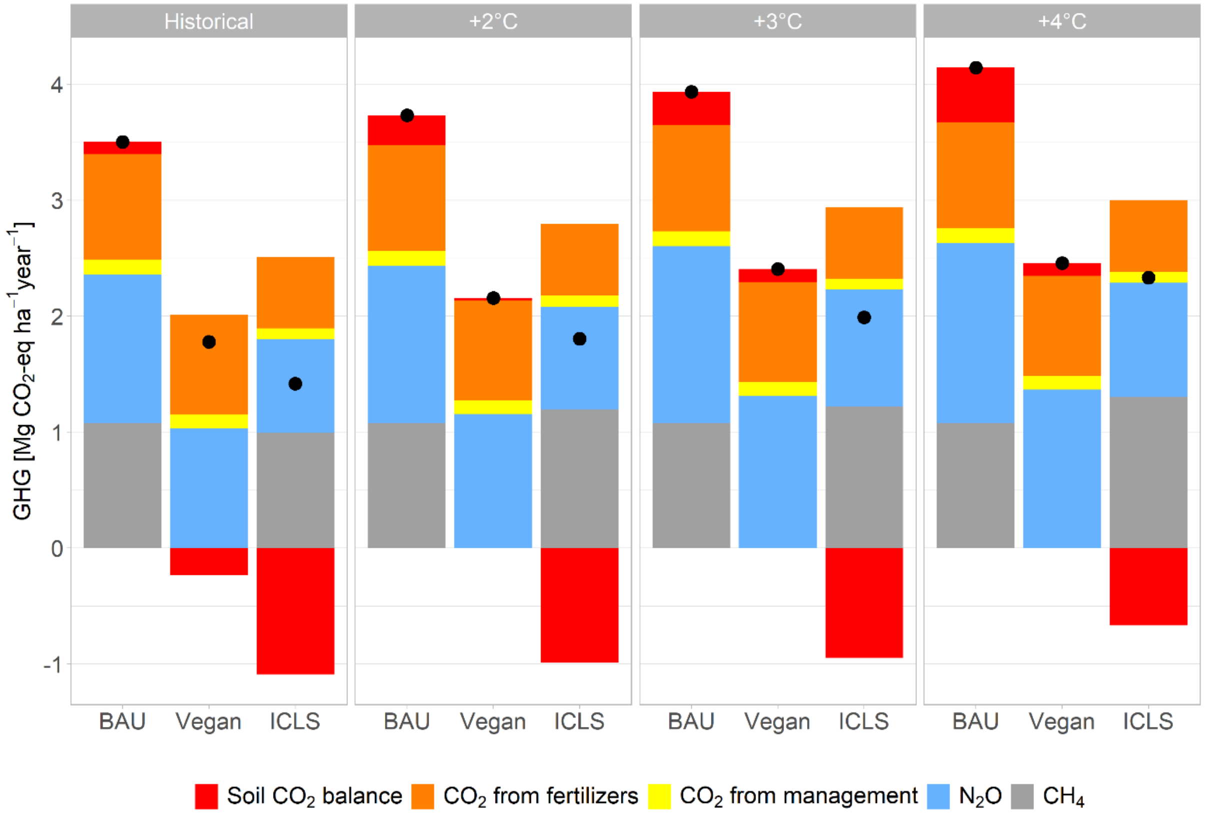

Figure S8. Greenhouse gas (GHG) emissions budget (CO_2, N_2O and CH_4; Mg CO_2-eq ha^{-1} year^{-1}). CO_2 from soil = SOC sequestration (negative) or release (positive); CO_2 from fertilizers = CO_2 emitted during N fertilizers manufacturing; CO_2 from management = CO_2 emitted due to fossil fuel use during agricultural management practices; N_2O = direct and indirect soil N_2O emissions, from the field and, within ICLS, also from manure storage; CH_4 = methane emissions resulting from the decomposition of manure spread on the field, and, within ICLS, also from sheep enteric fermentation and from manure storage (see Methods). Black points show net GHG budget of each scenario (sum of positive and negative emissions contributions). Global warming potential of GHG is computed as GWP (Supplementary Table S3). For climate change scenarios (+2°C, +3°C and +4°C), GHG budget is averaged between Earth System Models (MPI, CMCC and MIR).

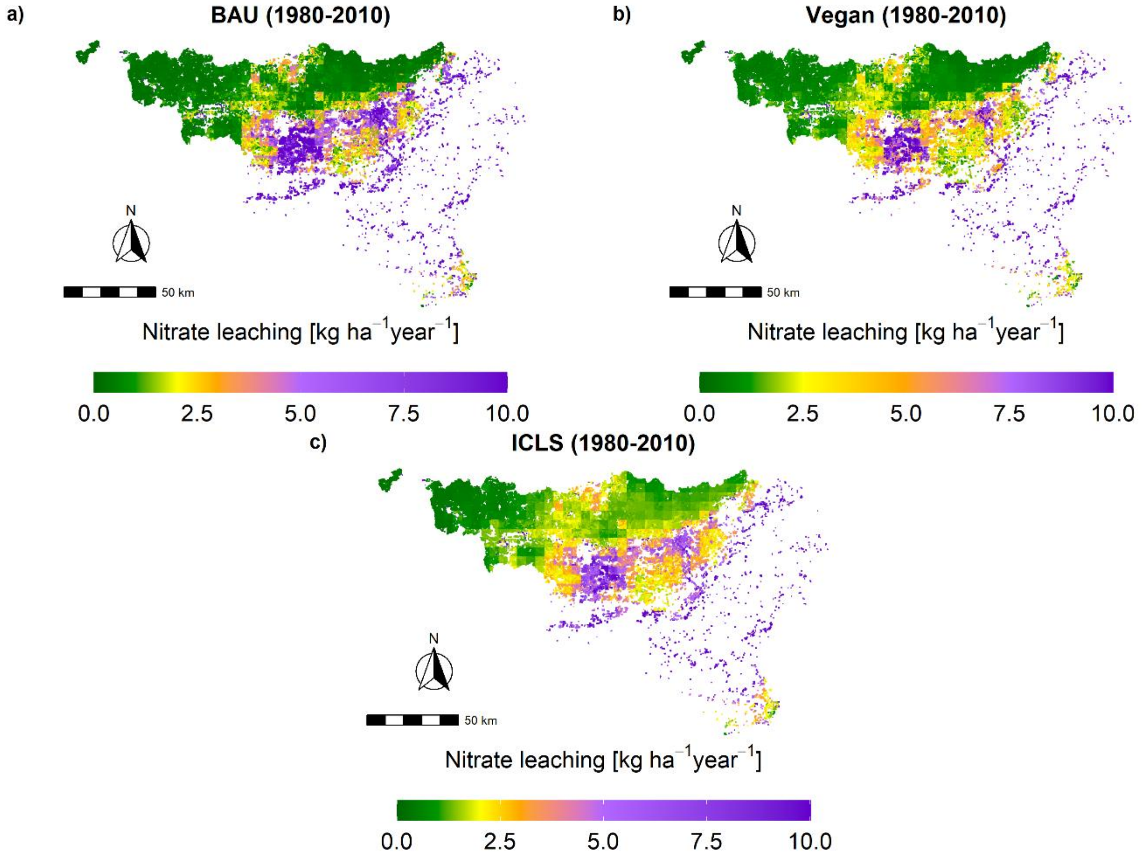

Figure S9. Nitrate leached at the base of the soil profile (below 2m depth) in historical climatic conditions (1980-2010), averaged over the 24-year simulation period across Wallonia, for the a) BAU, b) Vegan and c) ICLS scenarios.

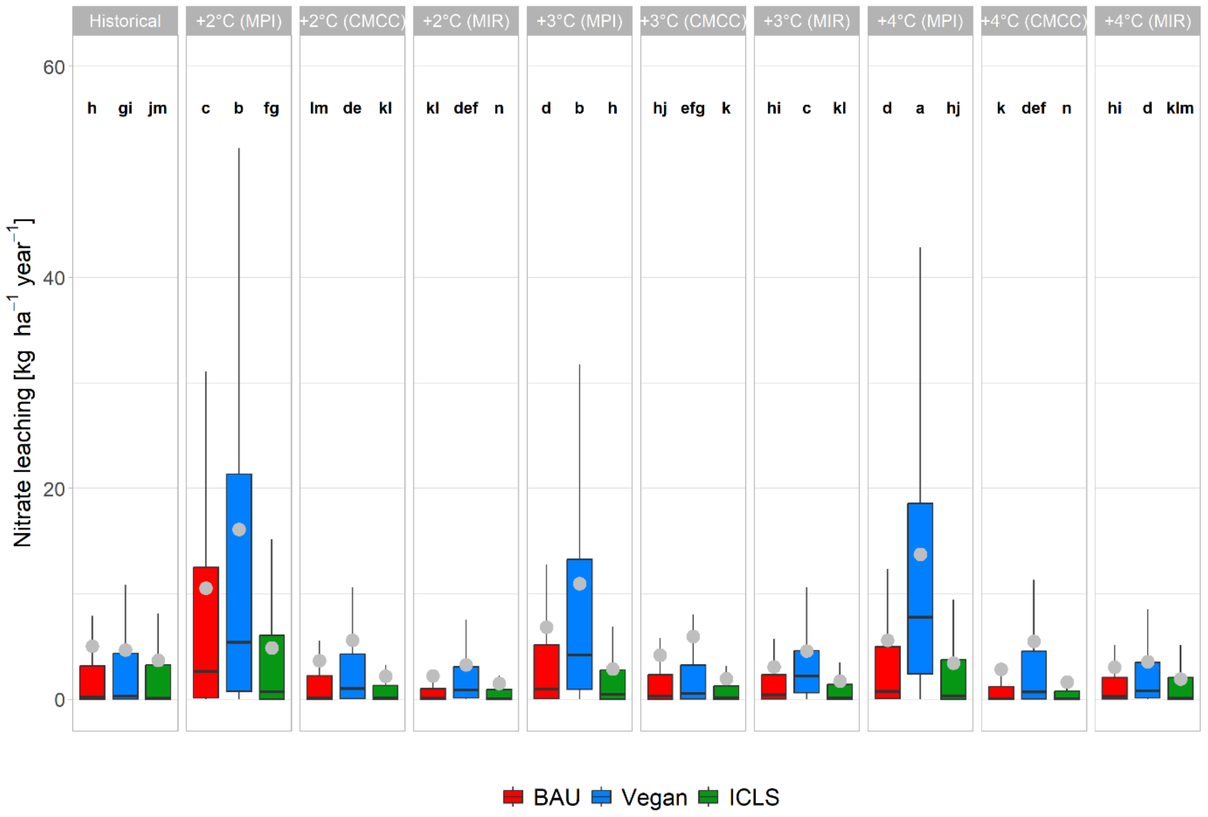

Figure S10. Nitrate leached at the base of the soil profile (below 2m depth) for the different circularity (BAU, Vegan, ICLS) and climate (historical, +2°C, +3°C, +4°C with the three Earth System Models (MPI, CMCC and MIR) scenarios, averaged over the 24-year simulation period across Wallonia. Boxplots show the interquartile range (25th – 75th percentiles), with minimal and maximal values displayed by vertical lines (outliers not shown). Horizontal lines and black circles show medians and means, respectively. Different letters above the boxplots denote statistically significant differences between groups as determined by Dunn's test (*p* < 0.05).

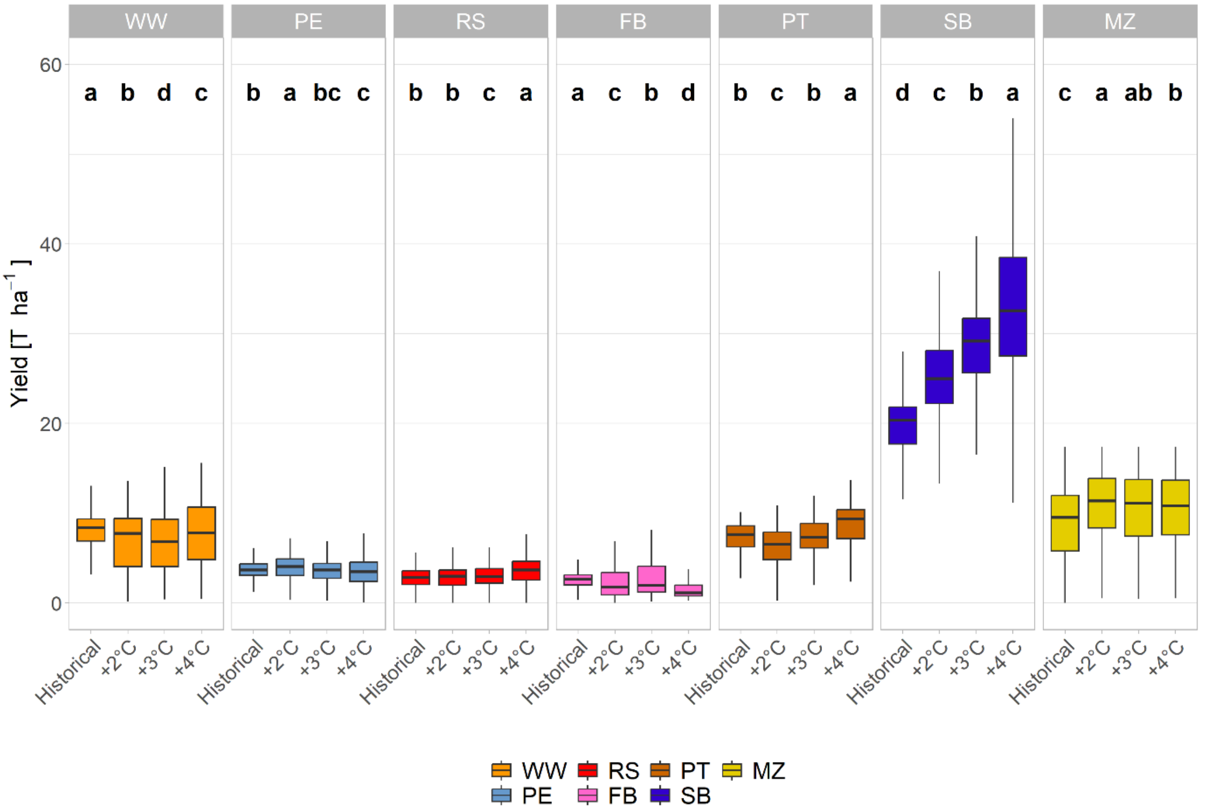

Figure S11. Crop yields distribution in Wallonia for the different climate scenarios (historical, +2°C, +3°C, +4°C), across circularity scenarios (BAU, Vegan, ICLS) and, for the climate change scenarios, across Earth System Models (MPI, CMCC and MIR). Boxplots show the interquartile range (25th – 75th percentiles), with medians displayed by horizontal lines and minimal and maximal values displayed by vertical lines (outliers not shown). Different letters above the boxplots denote statistically significant differences between groups as determined by Dunn's test (*p* < 0.05), respectively to each crop. WW = winter wheat; PE = pea; RS = rapeseed; FB = faba bean; PT = potato; SB = sugar beet; MZ = maize.

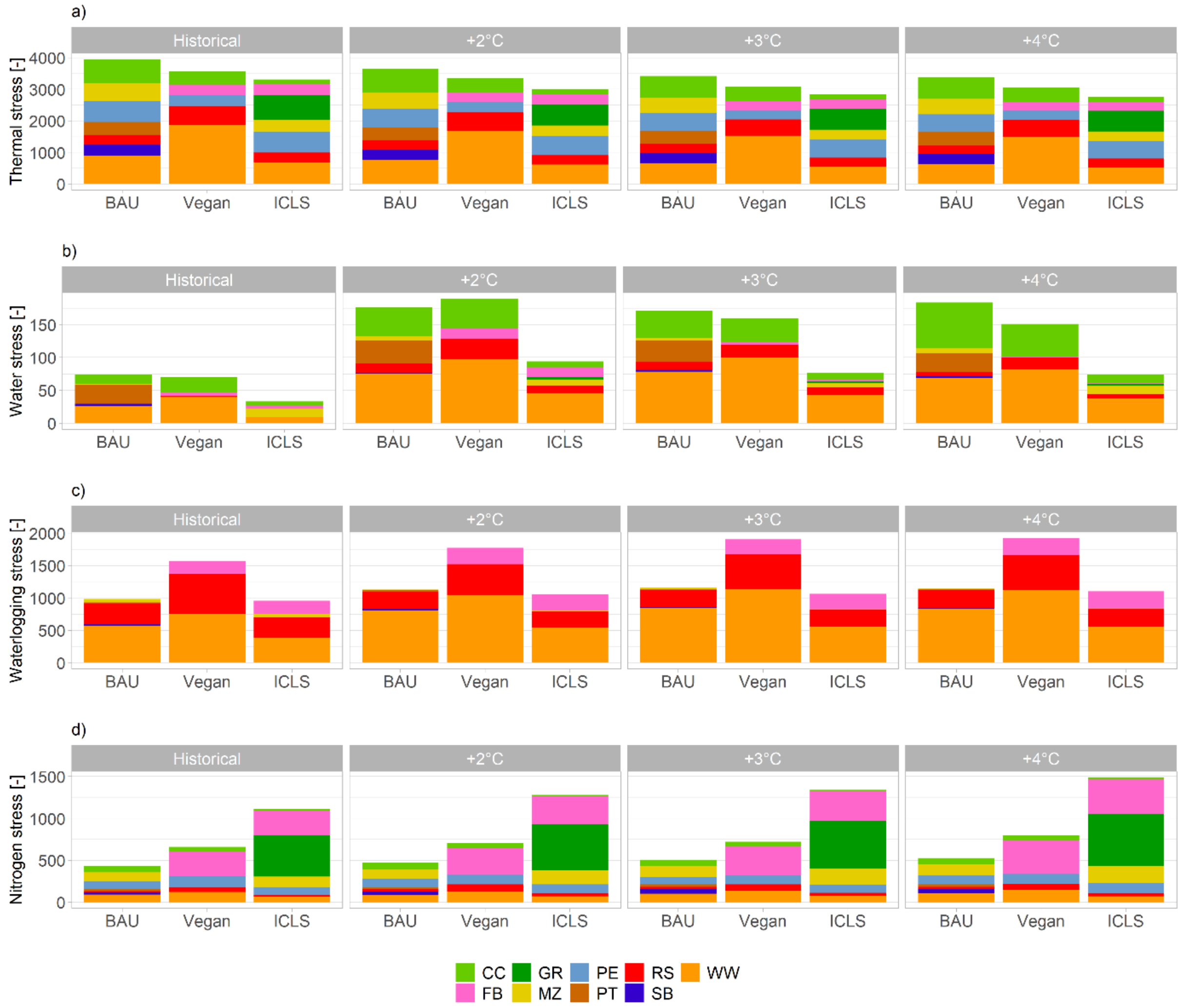

Figure S12. Crop stress evolution for the different circularity (BAU, Vegan, ICLS) and climate (historical, +2°C, +3°C; +4°C) scenarios across Wallonia. For the climate change scenarios (+2°C, +3°C, +4°C), stresses intensities are averaged between Earth System Models (MPI, CMCC and MIR). The crop stress intensity is defined as the area below the seasonal curves of the STICS variables a) *ftemp* (thermal stress), b) *swfac* (water stress), c) *exobiom* (waterlogging) and d) *inns* (nitrogen stress), these variables being comprised between 0 (maximal stress) and 1 (no stress) (Beaudoin et al., 2022). Crop stresses are summed up over an 8-year crop rotation, and then averaged over successive crop rotations and over UIDs in Wallonia. WW = winter wheat; RS = rapeseed; PE = pea; MZ = maize; FB = faba bean; SB = sugar beet; PT = potato; GR = grass.

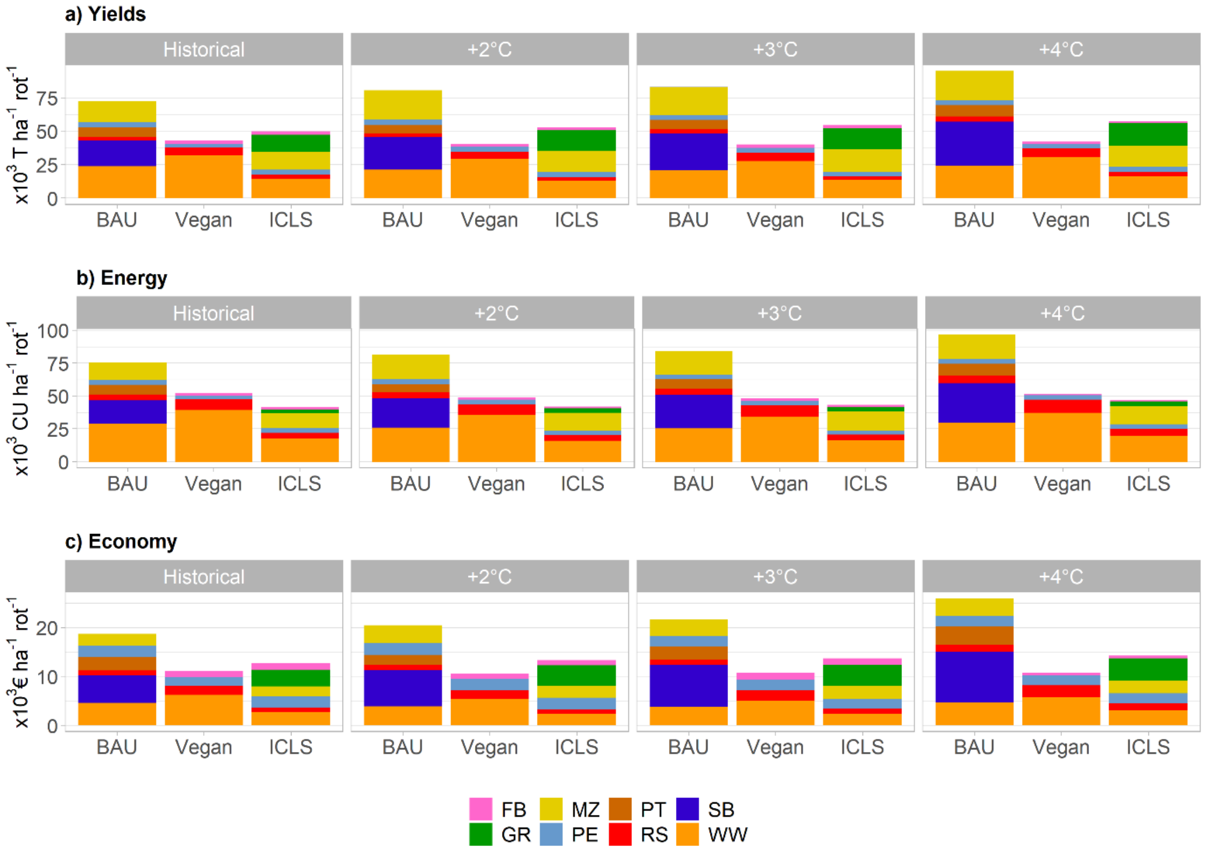

Figure S13. Overall productivity evolution across Wallonia for the different climate scenarios. Productivity is expressed as a) yield [tons], b) energetic value [cereal units], and c) economic value [euros]. For the climate change scenarios (+2°C, +3°C, +4°C), productivities are averaged between Earth System Models (MPI, CMCC and MIR). For economic metrics, grass also considers live weight gain. Metrics are detailed in Tables S1 and S2.

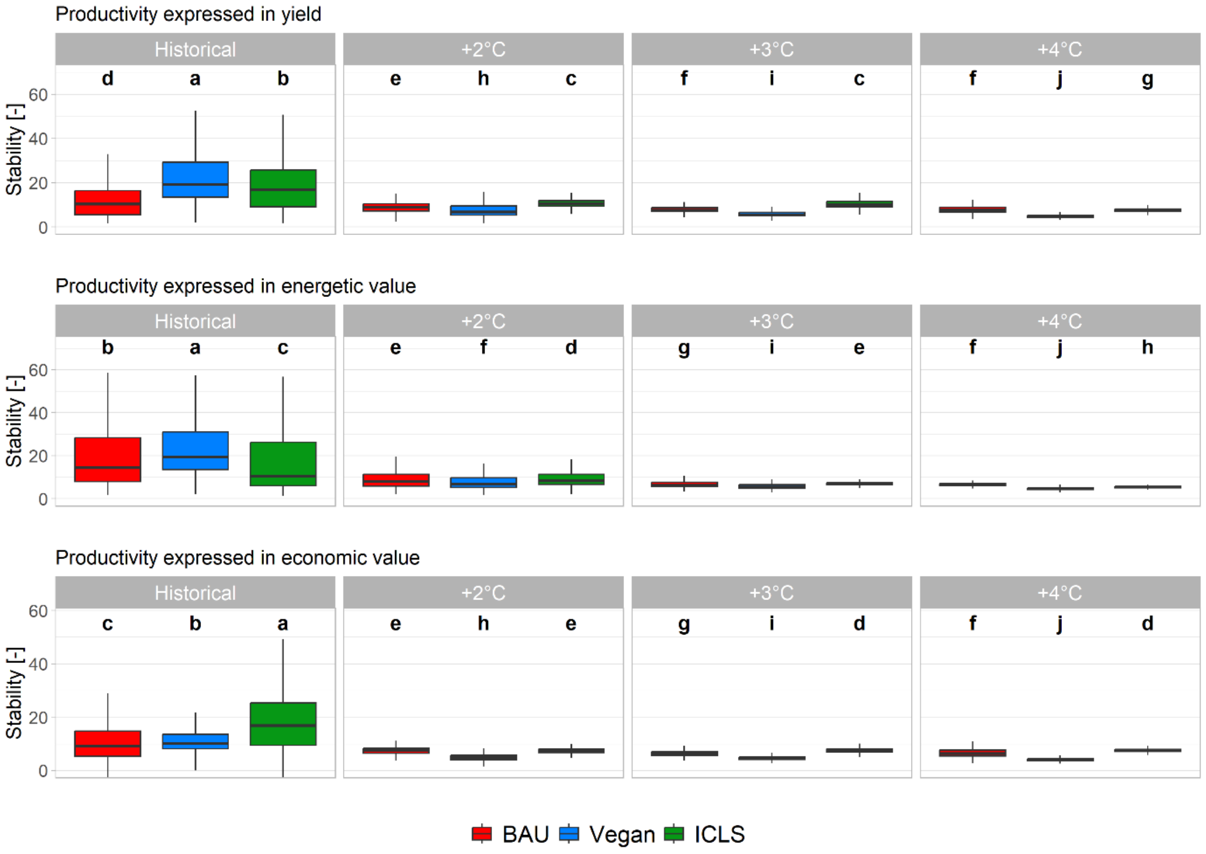

Figure S14. Productivity stability ($\frac{\mu}{\sigma}$, dimensionless), as the ratio of the mean productivity of a single 8-year crop rotation to its standard deviation over the 24-year simulation period (i.e., 3 successive rotations) across Wallonia for the circularity (BAU, Vegan, ICLS) and climate (historical, +2°C, +3°C, +4°C) scenarios. Stability is determined using a) yield [Mg] b) energy [cereal units] and c) economics [euros]. For the climate change scenarios (+2°C, +3°C, +4°C), stabilities are averaged between Earth System Models (MPI, CMCC and MIR). Boxplots show the interquartile range (25th – 75th percentiles), with medians displayed by horizontal lines and minimal and maximal values displayed by vertical lines (outliers not shown). Different letters above the boxplots denote statistically significant differences between groups as determined by Dunn's test ($p < 0.05$).

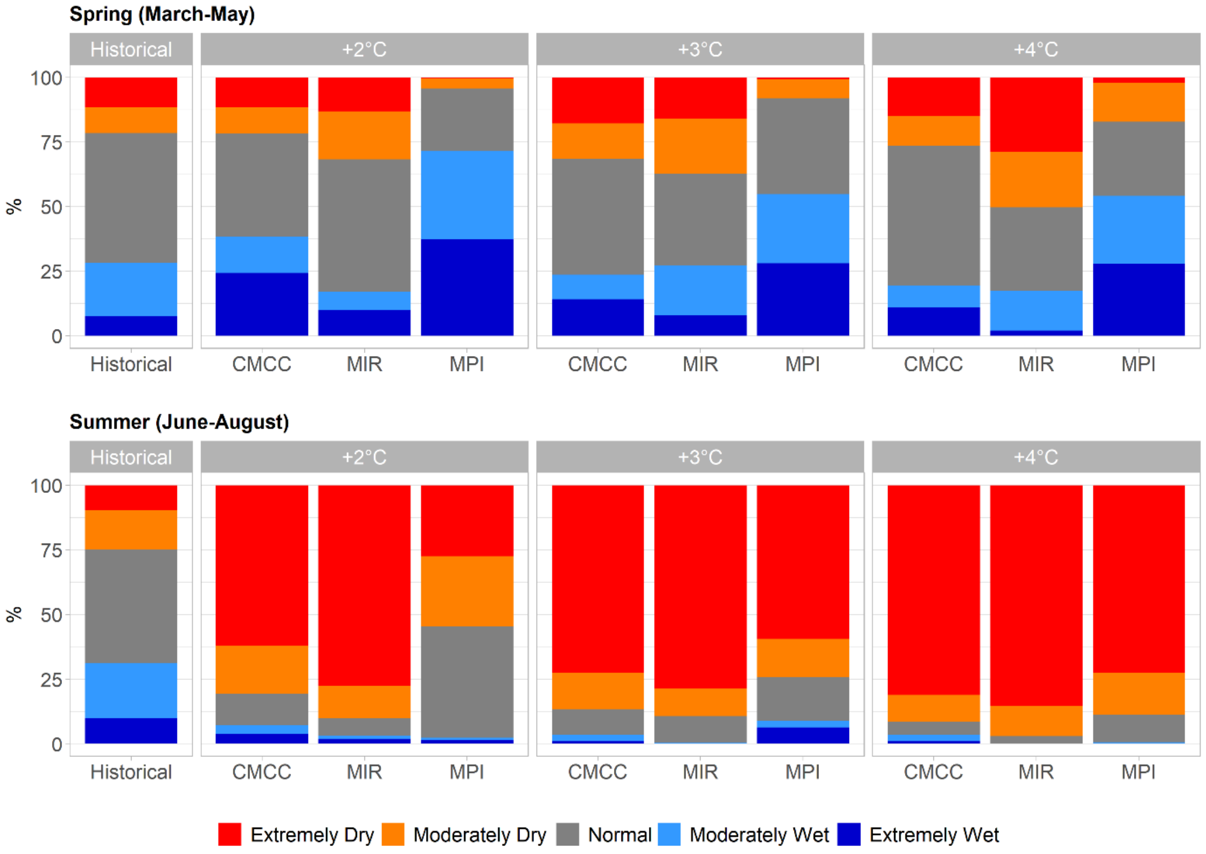

Figure S15. Percentage of climatic years, within each climate scenario and over Wallonia, which are classified as *normal* or *extremely/moderately dry/wet* based on the SPEI-3 drought index, which analyzes the water balance over 3-month periods (see Methods).

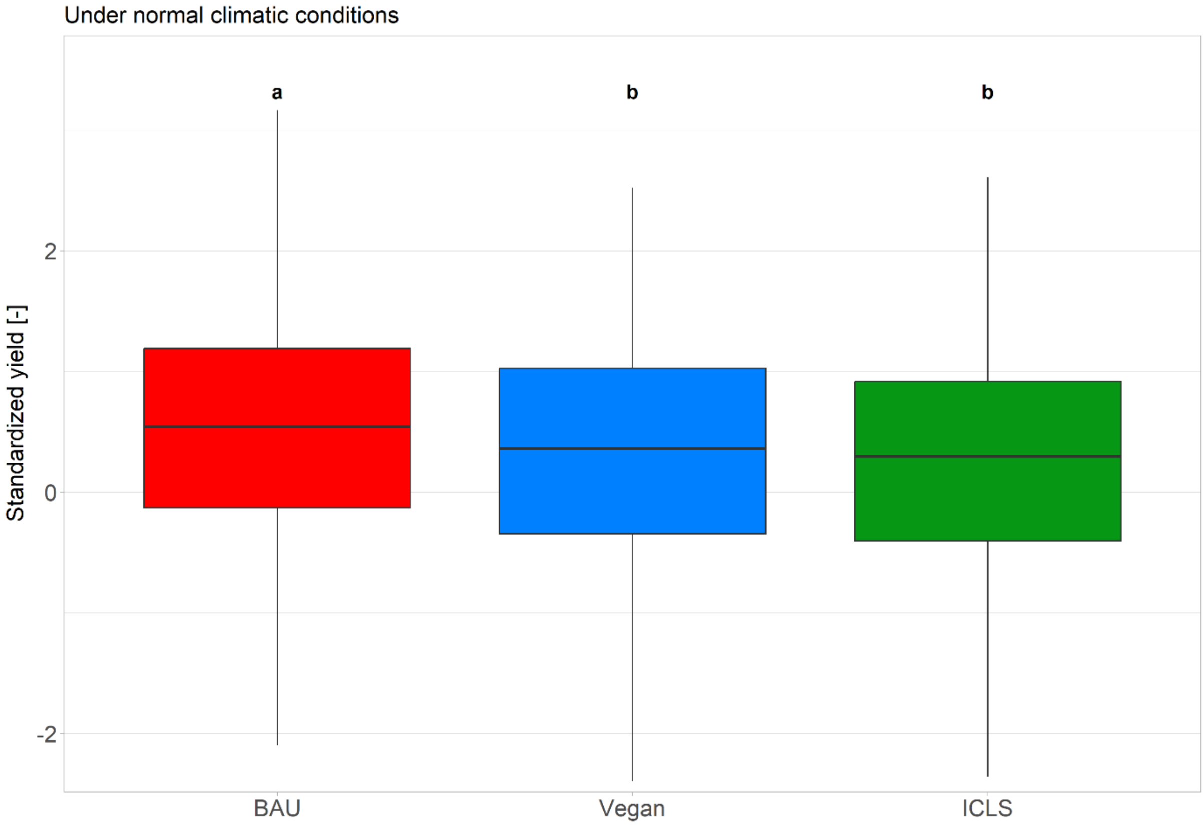

Figure S16. Wheat yields, standardized relatively to the UID and climatic and circularity scenarios, under *normal* climatic conditions as characterized by the SPEI-3 drought index (see Methods). Boxplots show the interquartile range (25th – 75th percentiles), with medians displayed by horizontal lines and minimal and maximal values displayed by vertical lines (outliers not shown). Different letters above the boxplots denote statistically significant differences between groups as determined by Dunn's test (p < 0.05).

B. Supplementary Tables

Crop	Conversion factor	Crop	Conversion factor
**Wheat**	1.04	**Sugar beet**	0.23
**Pea**	0.79	**Silage maize**	0.3
**Rapeseed**	1.3	**Potato**	0.22
**Faba bean**	0.54	**Grass**	0.61

Table S1. Cereal Unit conversion factors (from fresh weight in kg to cereal unit) for the different agricultural products (Brankatschk and Finkbeiner, 2014).

Commodity	Cost (€ ha^{-1})	Price (€ Mg^{-1})
**Wheat**	505	217
**Sugar beet**	920	86
**Potato**	2084	142
**Rapeseed**	559	444
**Pea**	320	562
**Faba bean**	390	567
**Silage maize**	459	151
**Sheep meat**	253	6650*

Table S2. Prices and costs used to compute productivity, averaged between 2018 and 2022. From Walloon Public Service (https://etat-agriculture.wallonie.be), Walloon Federation of Agriculture (https://federationwallonnedelagriculture.be), and the *Collège des Producteurs* (https://collegedesproducteurs.be). Costs include seeds, fertilizers, plant protection products, other allocated operating expenses, and subcontracted work. For pea, faba bean, silage maize and sheep meat, which concerned slightly different periods than 2018-2022, prices and costs have been adjusted using the yearly average inflation index in Belgium (Statbel, 2024). *For sheep meat, price is given per Mg of carcass weight.

Greenhouse gas	Lifetime (years)	GWP_{100}	GWP^*_{100}
**CO_2**	> 1000 years	1	1
**N_2O**	121	298	298
**CH_4**	12.4	34	7

Table S3. GWP and GWP* used for greenhouse gas emissions considered, at a 100-year time scale. Based on IPCC (2013), Cain et al. (2019), Smith et al. (2021), del Prado et al. (2021).

Crop	Sim. Yield (average; T ha^{-1})	Sim. Yield (standard deviation; T ha^{-1})	Obs. Yield from national statistics (average; T ha^{-1})
**Winter wheat**	9.2	2.6	8.8
**Sugar beet**	76.7	19.8	86.3
**Potato**	33.8	9.4	40.7
**Silage maize**	41.02	16.8	41.9
**Rapeseed**	3.2	1.2	3.73
**Pea**	4.4	1.4	3.4
**Faba bean**	3.04	1.06	4.2
**Temporary pasture**	6.4	1.6	8.35

Table S4. Yield statistics (in T ha^{-1}, fresh weight) from simulations under historical climatic conditions (1980-2010) across Wallonia, and actually produced in Wallonia between 2019 and 2023 (Statbel, 2024).

Indicator	Average simulated value	Observed trend	Reference for observations
**Soil organic carbon [0-30 cm]**	-0.23 Mg ha^{-1} year^{-1} for BAU	-0.3 Mg ha^{-1} year^{-1} in 1960-1990 for croplands in Wallonia	Lettens et al. (2005)
**Soil N_2O emissions**	2.43 (1.2-3.6) kg ha^{-1} year^{-1} for BAU	2.66 (1.92-3.4) kg ha^{-1} year^{-1} for BAU	Soil emission factor 1.8% (1.3-2.3%); Tian et al. (2020)
	1.85 (0.8-2.9) kg ha^{-1} year^{-1} for Vegan	2.52 (1.82-3.22) kg ha^{-1} year^{-1} for Vegan	
	1.44 (0.43-2.45) kg ha^{-1} year^{-1} for ICLS	1.8 (1.3-2.3) kg ha^{-1} year^{-1} for ICLS	
**Nitrate leaching**	5.05 kg N ha^{-1} year^{-1} for BAU	4 kg N ha^{-1} year^{-1}	Similar experiment in Northern France with cover crops, sugarbeet and wheat with close N fertilization levels; Yin et al. (2020)

Table S5. Comparison of average simulations in Wallonia under historical climatic conditions (1980-2010) and observed trends in Wallonia/Northern France for SOC, nitrous oxide (N_2O) emissions and nitrate leaching. References for observations are provided in the last column. For N_2O emissions, simulations are expressed as the median ($\pm$ the standard deviation), and for the observations, we compute the emissions expected from fertilization levels with soil emission factors.

C. Livestock and manure simulation

C.1. ICLS simulation

Integrated crop-livestock systems (ICLS) consist in crop rotations comprising 2.5 years of temporary pastures (Fig. 1c and S3). Sheep are nourished with the amount of biomass removed by cuts in temporary pastures and by cover crop. The calculation of these two feed quantities enables determining the number of sheep, which in turn allows for computing the amount of manure returned to the crops.

The sheep population in the ICLS is required to calculate the manure input for the 8-year ICLS rotation. The simulation was set up with an average stocking rate of 10 sheep per hectare. As one sheep is equivalent to 0.15 Livestock Unit (LU) (Benoit and Veysset, 2021), this represents a stocking density of 1.5 LU ha^{-1}, which is a common practice in Belgium with 32% of sheep farms having a stocking density between 1.6 and 3 LU ha^{-1} and 21% between 3 and 7 LU ha^{-1} (Peeters, 2010). On average, a single sheep produces 7.67 kg of fresh manure per day (Wybrecht et al., 2002).

$$Q = N * 7.67 * 365 \text{ [T ha}^{-1}\text{ rotation}^{-1}] \qquad (Eq. B1)$$

With the initial number of sheep of N = 10, we obtained an initial quantity of manure equal to 27.99 T ha^{-1} rotation^{-1}. This manure quantity enabled an initial simulation of an average 8-year ICLS. To refine our estimate of the number of sheep within the system, we considered that they are fed with grass biomass from pastures and cover crops. According to our initial simulations, the 8-year rotation produces x kg DM ha^{-1} of grass biomass from the pastures period and y kg DM ha^{-1} from cover crops, providing a total forage supply of $x+y$ kg DM ha^{-1}.

A sheep weighing 70 kg requires 0.8 kg of dry matter (DM) per day (Agabriel, 2010). We assumed that the 8-year rotation is continuously present on the farm, as farmers typically cultivate different crops from the rotation simultaneously across various field plots. Based on this, we determined the ICLS carrying capacity as follows:

$$N = \frac{x + y}{8 * 0.8 * 365} \text{ [sheep ha}^{-1}] \qquad (Eq. B2)$$

ICLS stocking density and the subsequent manure fertilization were adapted for each climate

scenario as function of pastures and cover crop average biomass, for each of the 11,515 UIDs in Wallonia, according to Equations B1 and B2.

Sheep live weight gain is computed from the amount of grass removed by cuts, simulating dry matter intake (DMI), using a forage conversion ratio equal to 0.1 kg LW gain per kg DMI (Savian et al., 2014). Following the methodology of Delandmeter et al. (2024a), the quantity of sheep meat is determined considering (*i*) a dressing percentage, i.e. the ratio of carcass weight over total live weight, equal to 46%, and (*ii*) that, in average, 80% of this carcass is available for retail product (Gérard et al., 2022). Methane (CH_4) emissions produced from enteric fermentation are estimated as 2% of DMI (Savian et al., 2014).

C.2. GHG budget computation linked to manure

ICLS scenario

CH_4 emissions resulting from the decomposition of manure occur during both manure storage and spreading on the field. Since sheep manure is stored as solid before being applied to pastures, it typically decomposes under more aerobic conditions, resulting in lower CH_4 emissions (IPCC, 2006). Therefore, as it does not represent a significant share of emissions, following the procedure of IPCC (2006), we base on the Tier 1 approach which provides average methane emissions due to manure as equal to 1 kg CH_4 head^{-1} year^{-1} given the average annual temperatures being between 11°C and 14°C for all climatic scenarios.

N_2O emissions also originate from manure storage, either directly through nitrification (aerobic conditions) and denitrification (anaerobic conditions), or indirectly through volatile nitrogen losses. These emissions are computed following the Tier 1 approach of IPCC (2006). Direct N_2O emissions are computed as 0.5% of nitrogen excreted (IPCC, 2006). Indirect N_2O emissions are based on the fraction of volatilized nitrogen, estimated as 12% of nitrogen excreted, and on the fraction of N_2O in this volatilized nitrogen, estimated as 0.01 kg N_2O-N (kg NH_3-N + NO_x-N volatilized)$^{-1}$ (IPCC, 2006).

BAU scenario

In the BAU scenario, we only consider CH_4 emissions resulting from the anaerobic

decomposition of manure spread on the field. Based on Phan et al. (2012), which measured, using a closed chambre technique, CH_4 fluxes originating from the spreading on surface of 40 T ha^{-1} of solid beef cattle manure – exactly as practiced in the BAU system, these emissions are estimated to be equal to 252 kg CH_4 ha^{-1} for all climatic scenarios.

N_2O and CH_4 emissions linked to manure storage were not considered for the BAU scenario, as we do not perform here a LCA but compute the GHG budget at the scale of the farm, and as such storage occurs outside the farm in the BAU scenario.

D. Weather data

Originally, the three Earth System Models (ESMs) MPI, CMCC and MIR predict temperature increases in different 30-year periods, meaning that they do not lead to the exact same CO_2 atmospheric concentrations for the same global warming rate (+2°C, +3°C and +4°C). In order to avoid a bias due to different CO_2 atmospheric concentrations, we uniformized this concentration within each scenario by (*i*) computing the average CO_2 concentration over the 30-year period for the MPI model (which has periods comprised between the periods of CMCC and MIR models) and (*ii*) using this average concentration for all three ESMs MPI, CMCC and MIR (Table S6). This methodology creates, for each 30-year period of +2°C, +3°C and +4°C global warming rate, three sets of climatic data which only differ by temperatures and pluviometry, and hence, allows to compare the influence of contrasting climatic conditions without any bias due to e.g. CO_2 fertilization effect.

		**Scenario**					
**ESM**	**RCM**	**+2°C**		**+3°C**		**+4°C**	
		CO_2 content [ppm]	SSP scenario	CO_2 content [ppm]	SSP scenario	CO_2 content [ppm]	SSP scenario
MPI-ESM1-2-HR **(MPI)**	MARv3.14	475	SSP370	627	SSP370	1006	SSP585
CMCC-CM2-SR5 **(CMCC)**	MARv3.14	475	SSP370	627	SSP370	1006	SSP585
MIROC6 **(MIR)**	MARv3.14	475	SSP370	627	SSP370	1006	SSP585

Table S6. Future climate scenarios details.

E. STICS inputs and outputs data

All the inputs required to run the STICS model (soil, climate, plant and management data formatted for STICS), as well as the corresponding model outputs for all scenarios investigated in this study, are available in the following shared repository: https://doi.org/10.6084/m9.figshare.31293703.

References for Supplementary Material

Agabriel, J. (2010). Alimentation des bovins, ovins et caprins. Besoins des animaux-Valeurs des aliments: Tables Inra 2010. Édition remaniée. Editions Quae.

Benoit, M., & Veysset, P. (2021). Livestock unit calculation: a method based on energy needs to refine the study of livestock farming systems.

Diles, J. J. B., Green, R. D., Shepard, H. H., Mathiews, G. L., Hughes, L. J., & Miller, M. F. (1996). Relationships between body measurements obtained on yearling brangus bulls and measures of carcass merit obtained from their steer clone-mates. *The Professional Animal Scientist*, *12*(4), 244-249.

Gérard, A., Rainon, C., Solas, L., Legroux, L., Chevalier, A., Miquel, M. (2022). Référentiel technico-économique ovin viande. Available in https://idele.fr/inosys-reseaux-elevage

IPCC. (2006). Emissions from livestock and manure management. In 2006 IPCC Guidelines for National Greenhouse Gas Inventories (Vol. 4, Agriculture, Forestry and Other Land Use, Chapter 10). Institute for Global Environmental Strategies (IGES), Hayama, Japan.

IPCC. (2013). Climate Change 2013: The Physical Science Basis. Contribution of Working Group I to the Fifth Assessment Report of the Intergovernmental Panel on Climate Change (T. F. Stocker, D. Qin, G.-K. Plattner, M. Tignor, S. K. Allen, J. Boschung, A. Nauels, Y. Xia, V. Bex, & P. M. Midgley, Eds.). Cambridge University Press.

Lettens, S., Van Orshoven, J. O. S., Van Wesemael, B. A. S., Muys, B., & Perrin, D. (2005). Soil organic carbon changes in landscape units of Belgium between 1960 and 2000 with reference to 1990. *Global Change Biology*, *11*(12), 2128-2140.

Peeters, A. (2010). Country Pasture/Forage Resource Profiles'. *Food and agriculture organization: Rome (Italy)*.

Phan, N. T., Kim, K. H., Parker, D., Jeon, E. C., Sa, J. H., & Cho, C. S. (2012). Effect of beef cattle manure application rate on CH4 and CO2 emissions. *Atmospheric environment*, *63*, 327-336.

Savian, J. V., Neto, A. B., de David, D. B., Bremm, C., Schons, R. M. T., Genro, T. C. M., ... & de Faccio Carvalho, P. C. (2014). Grazing intensity and stocking methods on animal production and methane emission by grazing sheep: Implications for integrated crop–livestock system. *Agriculture, ecosystems & environment*, *190,* 112-119.

Tian, H., Xu, R., Canadell, J. G., Thompson, R. L., Winiwarter, W., Suntharalingam, P., ... & Yao, Y. (2020). A comprehensive quantification of global nitrous oxide sources and sinks. *Nature*, *586*(7828), 248-256.

Wilkinson, J. M. (2011). Re-defining efficiency of feed use by livestock. *animal*, *5*(7), 1014-1022.

Wybrecht, B., Paquot, E., Leplaideur, M.-A., Williams, D., Fovet-Rabot, C., Loubet, M. (2002). Memento de l'agronome. Centre de Coopération Internationale en Recherche Agronomique pour le Développement, Montpellier, France.
